# Interpenetrating polymer networks for desalination and water remediation: a comprehensive review of research trends and prospects†

**DOI:** 10.1039/d2ra07843k

**Published:** 2023-02-20

**Authors:** Soumi Dutta, Ria Sen Gupta, Shabnam Pathan, Suryasarathi Bose

**Affiliations:** a Department of Materials Engineering, Indian Institute of Science Bengaluru 560012 India sbose@iisc.ac.in

## Abstract

Interpenetrating polymer network (IPN) architectures have gained a lot of interest in recent decades, mainly due to their wide range of applications including water treatment and environmental remediation. IPNs are composed of two or more crosslinked polymeric matrices that are physically entangled but not chemically connected. In polymer science, the interpenetrating network structure with its high polymer chain entanglement is commonly used to generate materials with many functional properties, such as mechanical robustness and adaptable structure. In order to remove a targeted pollutant from contaminated water, it is feasible to modify the network architectures to increase the selectivity by choosing the monomer appropriately. This review aims to give a critical overview of the recent design concepts of IPNs and their applications in desalination and water treatment and their future prospects. This article also discusses the inclusion of inorganic nanoparticles into traditional polymeric membrane networks and its advantages. In the first part, the current scenario for desalination, water pollution and conventional desalination technologies along with their challenges is discussed. Subsequently, the main strategies for the synthesis of semi-IPNs and full-IPNs, and their relevant properties in water remediation are presented based on the nature of the networks and mechanism, with an emphasis on the IPN membrane. This review article has thoroughly investigated and critically assessed published works that describe the latest study on developing IPN membranes, hydrogels and composite materials in water purification and desalination. The goal of this critical analysis is to elicit fresh perspectives regarding the application and advantages of IPNs in desalination and water treatment. This article will also provide a glimpse into future areas of research to address the challenges relating to advanced water treatment as well as its emerging sustainable approaches. The study has put forward a convincing justification and establishes the relevance of IPNs being one of the most intriguing and important areas for achieving a sustainable generation of advanced materials that could benefit mankind.

## Abstract 2

A comprehensive review of research trends and prospects for interpenetrating polymer networks (IPNs) for desalination and water remediation is presented in this article.

## Graphical abstract

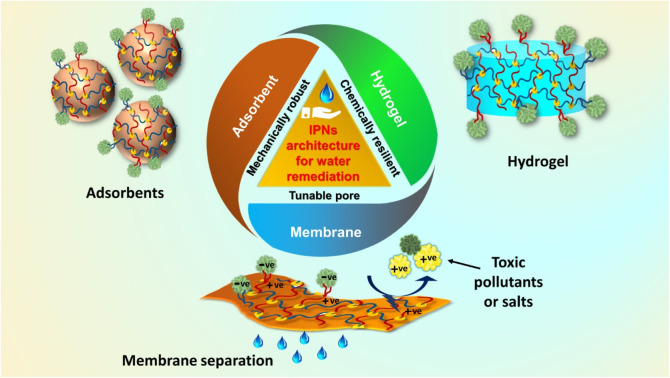

## 1. Background and overview

**Fig. 1:**
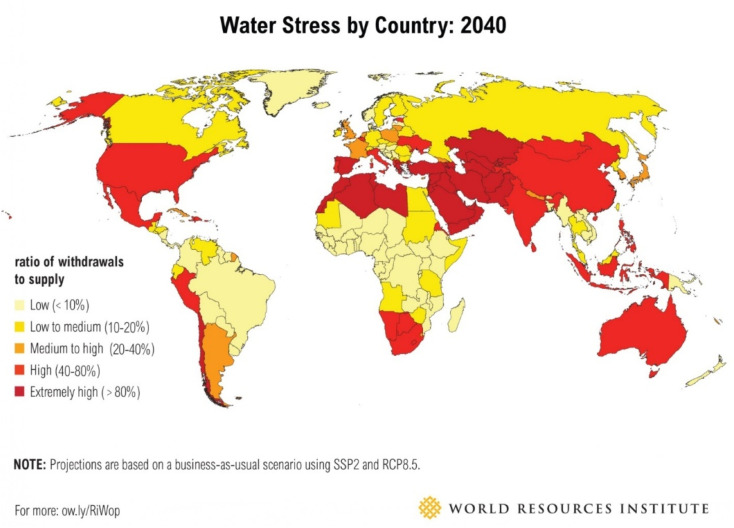
Predicted water stress in the world by 2040 (this figure has been reproduced from ref. 6 with permission from World Resources Institute, copyright 2015).

Water is a precious natural resource that is essential for life to survive on earth. To address the rising demand for water supply due to industrialization and urbanization, the world is currently facing a water quality and quantity crisis.^1–4^ Although groundwater is a significant source of clean drinking water, it is frequently contaminated in many regions of the world. Freshwater has been incessantly polluted by various activities such as rapid industrialization, the fast growth of the population, and agricultural activities. The severity of this problem is also made more difficult by the deterioration of groundwater levels and the contamination of water sources. Even though the earth is more than 70% covered in water, much of it is too salty to be useful. Freshwater makes up only 3% of the total water mass, and very little of it is easily accessible. This allows for the preservation of the majority of the freshwater in frozen glaciers or underground water reservoirs. In addition, water pollution is considered one of the major threats to human health and aquatic bodies. On the other hand, utilizing unconventional water sources, such as rainwater, tainted freshwater, brackish water, industrial effluent, and seawater has become the norm due to increasing strain on the world's water supply, especially in traditionally water-stressed regions.^5^Fig. 1 shows the anticipated water stress worldwide by 2040 (adapted from Luo *et al.*^6^).

Desalination is one of the key solutions to this challenge since it allows for the reuse of seawater, which is the most abundant source of water for both industrial usage and human consumption. The removal of salts and minerals from seawater to generate freshwater is referred to as desalination. The process of desalinating involves eliminating salt from water Thomas Jefferson first came up with the idea in 1791.^7,8^ Both brackish and seawater are typically treated by desalination. Commonly, salts like NaSO_4_, NaCl, MgCl_2_, LiCl, and MgSO_4_ are eliminated during the desalination procedure. However, the excessive salt of seawater makes it unsuitable for residential use. In ancient times, evaporative desalination technology was employed for removing salt from seawater. Indeed, it has long been employed to supply clean drinking water in many desolate and rural regions, as well as coastal areas. Although conventional water treatment procedures are successful at removing nutrients and organic debris, they are less effective in reducing water salinity. In this instance, advanced desalination technologies are a viable alternative that may bridge the large gap between current capabilities and rising demand.^5^

In the twenty-first century, the discovery of nanomaterials is the primary driving force behind technological and industrial advancement. This led to considerable improvements in understanding and breakthroughs in environmental remediation, especially in radically altering traditional water treatment processes that have been in use for a century. Engineered nanomaterials will undoubtedly have an influence on water treatment practice in the future since they open up new pathways and offer a plethora of opportunities to explore their impact in several disciplines.

Membrane technology accounts for up to 53% of all technologies used globally to provide clean water due to its ease of use, low cost, lack of phase change, high productivity, ease of scale-up, and high separation efficiency.^9^ It is significant in the treatment of brackish and wastewater, desalination of seawater (its reuse for consumption), the dairy sector for milk skimming and effluent treatment, and many other applications.^10,11^ This cutting-edge technology is crucial in the treatment of brackish and wastewater, desalting of seawater (reuse for drinking purposes), the dairy sector for milk skimming, and industrial effluent treatment, among other applications. A membrane is a physical barrier that permits only desired elements of a liquid to flow through while undesirable ones are retained on the membrane surface depending on membrane characteristics like pore size or charge. There are primarily two types of membranes: polymeric membranes and inorganic membranes. Metal or ceramic-based inorganic membranes provide a high degree of structural, mechanical, and thermal resilience. Despite their low permeability and great selectivity, they are less desirable for a variety of applications.^10,12^ Contrarily, polymeric membranes are frequently used in pressure-driven filtration systems, such as reverse osmosis (RO), ultrafiltration (UF), and nanofiltration (NF), due to their superior flexibility, excellent film-forming properties, tensile stability, chemical resistance, high tunability, specific transmit of the chemical compound, and separation processes. For the fabrication of polymeric membranes, materials like polyvinyl alcohol (PVA), polyether sulfone (PES), polyvinylidene fluoride (PVDF), polyvinyl chloride (PVC), polypropylene (PP), polyacrylonitrile (PAN), polyimide (PI), polyethylene (PE), cellulose acetate (CA), polyamide (PA), and chitosan are typically used.^9,10^ Additionally, the membrane propriety such as pore size, selectivity, and charge of a polymeric membrane is adjustable by altering the casting conditions, monomer molecules, doses, additives, and coagulation bath conditions. However, polymeric membranes also have several serious disadvantages, including limited chlorine resistance and susceptibility to fouling because of their innate hydrophobicity.^13^ Depending on the level of fouling, a thorough physical and chemical cleaning procedure may be necessary, or the membrane may need to be replaced frequently. Another challenge is the significant trade-off between selectivity and permeability; it is challenging to enhance one without compromising the other. These flaws were addressed by physical and chemical modifications to the polymer chain.

Interpenetrating polymer networks (IPNs) have received a lot of interest in numerous studies.^14–17^ It consists of two or more polymer chains, each in network form, at least one of which is synthesized and/or cross-linked in the vicinity of the other without covalently bound to one another. The network is inseparable unless the chemical linkages are broken. In addition to the enhanced chemical architecture of the macromolecular matrix, IPN represents a revolutionary advancement in polymeric science due to its tunable pore size and charge density to satisfy various requirements. Further, it offers mechanical, chemical, and thermal stability over a wide range of environmental conditions. These systems also called “hungry networks” or “intelligent polymers”, are now the focus of intensive scientific research because of their potential for technological use in several disciplines, including healthcare, industry, biology, and environmental remediation.^18^ The use of IPN is not only confined to membrane applications; it also extends to adsorbent and hydrogel synthesis in a variety of fields. In this article, we will emphasize on applications for water treatment and desalination using IPN-based membranes and other compounds.

The IPN is a type of polymeric structure that essentially consists of two or more physically interlaced polymer networks without any covalent bonds between or among them.^19^ Taking these advantages into consideration, this approach is utilized to modify the structure of polymeric membranes and adsorbents, where the polymer grows from the primary polymer backbone through conventional polymerization.^20^ The IPN structure makes the membrane more effective in terms of mechanical robustness, tunable pore creation, chemical resilience, and antifouling qualities, in comparison to a standard blend membrane.^21^

**Fig. 2:**
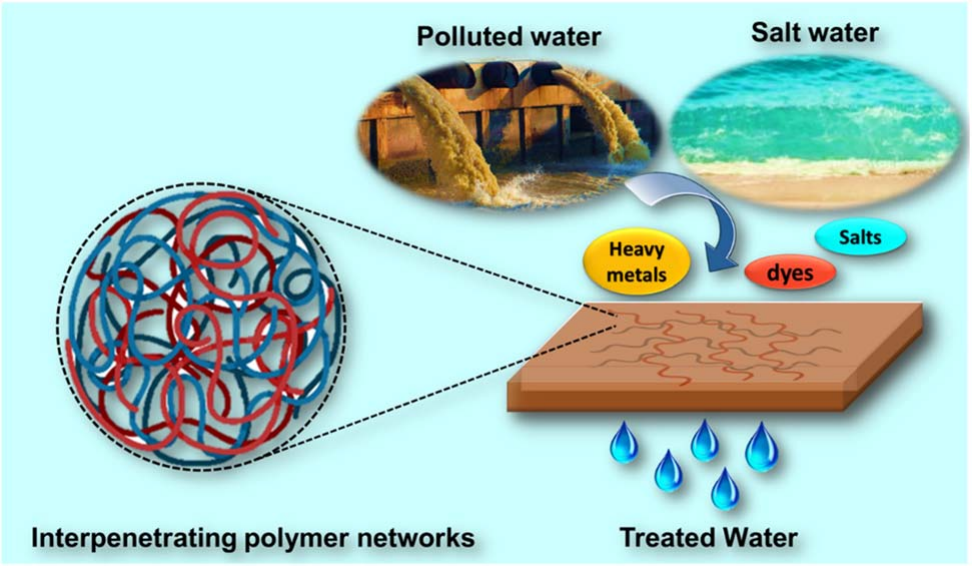
Schematic representation of water remediation using interpenetrating polymer network membrane technology.

This review article focuses on the most recent advancements in IPN concept, their usage in water treatment and desalination as schematically depicted in Fig. 2, and its implication for the future. In the first part, the current scenario of conventional water treatment and desalination technologies and their challenges is discussed. Followed by, the basic IPN classification will also be covered to better understand the importance of IPN architecture and its advantages. In the second part, an overview of the most specific applications of IPN in separation processes including desalination, dye rejection, and removal of hazardous pollutants including heavy metals, pharmaceutical, antibacterial properties, and other water remediation applications is critically presented based on the recently published articles. The review article will also elaborate on the plausible mechanism for water treatment using IPNs, as well as emerging sustainable approaches for cutting-edge water treatment technology. This review will conclude by outlining potential areas for further research into IPN-based water purification.

## 2. Interpenetrating polymer networks (IPNs): definition, advantages, classifications

**Fig. 3:**
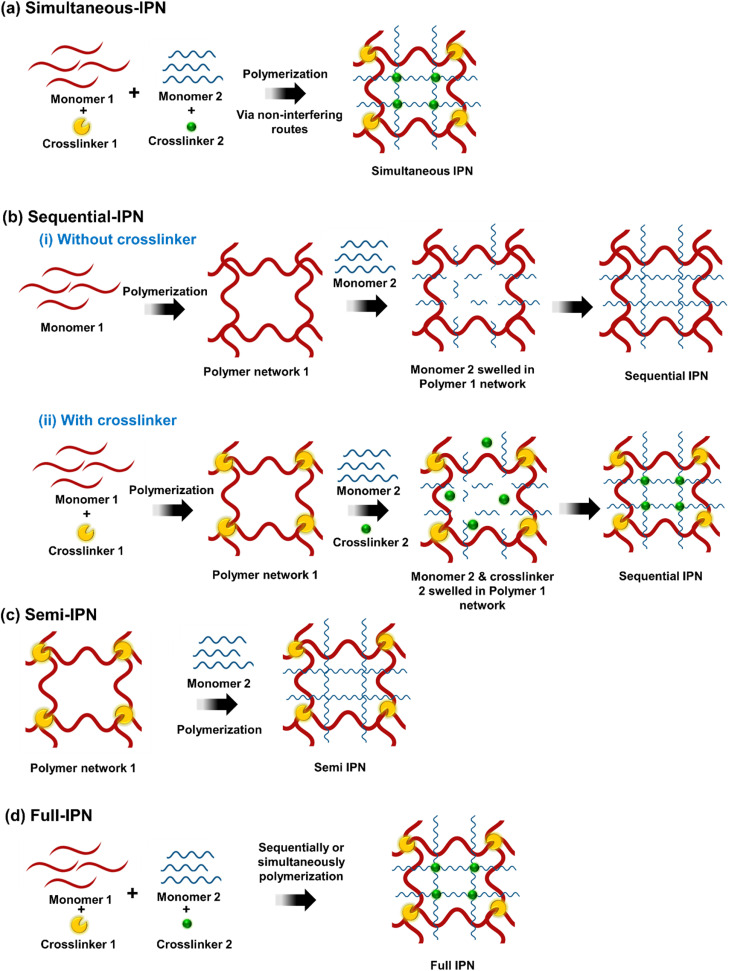
Schematic representation of Interpenetrating Polymer Networks (IPNs) synthesis process: (a) simultaneous-IPN, (b) sequential-IPN, (c) semi-IPN, (d) full-IPN.

The term “interpenetrating polymer networks” (IPNs) refers to a combination of two or more polymers that are synthesized in immediate contact with one another and are linked in network form. IPN refers to a form of polymer where two chemically separate networks coexist, with a structure that is ideally homogeneous down to the segmental level, such that there is some sort of “interpenetration”.^16,22^ According to their chemical bonding features, pattern of rearrangement, and synthesis process, IPNs are divided into several categories mainly simultaneous-IPN, sequential-IPN, full-IPN, and semi-IPN. IPNs can be classified into semi-IPNs and full-IPNs based on whether one or both of the relevant components are cross-linked.^16,17,23^ All possible IPN structures are depicted schematically in Fig. 3.

### 2.1. Simultaneous-IPN

The crosslinkers and activators from both networks are combined with the monomers or prepolymers in simultaneous-IPN. Although they occur simultaneously, the two network chains don't interfere with one another (Fig. 3a).

### 2.2. Sequential-IPN

In sequential-IPNs, a polymeric network is synthesized one after the other; the crosslinker could be present or absent as illustrated in Fig. 3b. In the first instance, monomer 1 is first polymerized, then monomer 2, resulting in the successive formation of a network without the need of a crosslinker. As seen in Fig. 3b(i). In the second case, monomer 1 and crosslinker 1 are used to create the first, polymer network 1. The second interpenetrating polymeric network is generated *in situ* after monomer II and crosslinker 2 are swelled into network I, as shown in Fig. 3b(ii).

### 2.3. Semi-IPN

In the semi-IPN only one polymeric chain assembly is crosslinked, leaving the other in linear form (Fig. 3c).

### 2.4. Full-IPN

The full-IPN is comprised of two or more polymeric networks that are individually interlocked through crosslinkers but not covalently connected to one another and can only be separated by breaking chemical bonds. These can be made in either sequential or simultaneous processes (Fig. 3d).

IPNs architecture has gained great attention in the last decades, mainly due to its wide range of applications including water treatment and environmental remediation applications. This review aims to give an overview of the recent design concepts of IPN and their applications in water treatment and desalination and their future aspect.

## 3. Desalination and water treatment using traditional treatment technologies: a brief overview

**Fig. 4:**
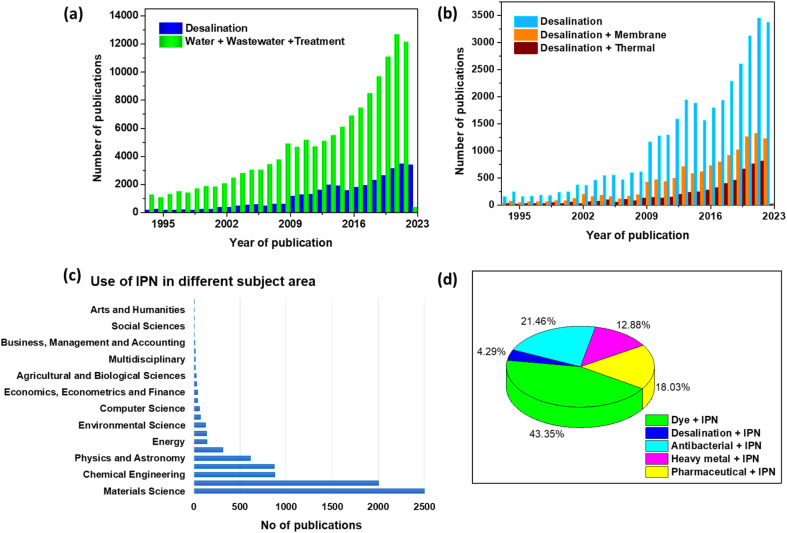
The number of publications in the last 30 years related to (a) desalination and water and wastewater treatment, (b) desalination and desalination *via* membrane and thermal technology, (c) use of IPN in different subject areas, (d) use of IPN in different pollutant removal (Scopus database with the keywords “mentioned in the plot” as of 11th November 2022).

**Fig. 5:**
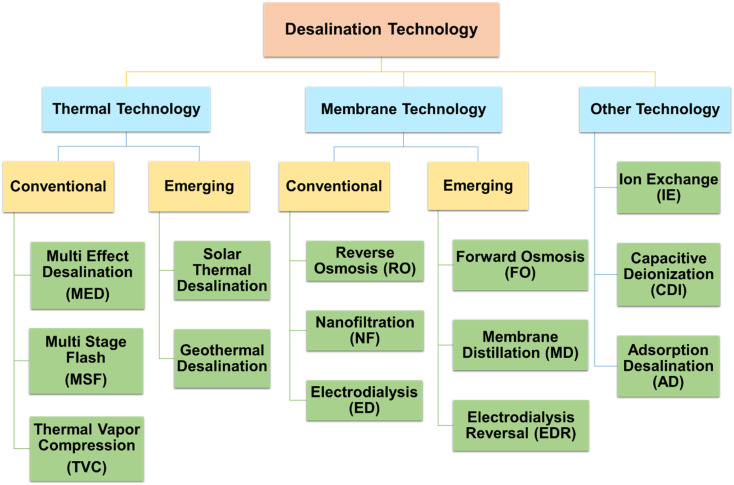
Broad classification of desalination technology.

Desalination, also known as desalting, is the process of removing dissolved salts from seawater, brackish water, municipal wastewater, and even high-mineral-content groundwater.^24^ Desalination and wastewater water treatment for reuse are now the two most significant approaches to reducing water scarcity to meet the demands of increasing population, economic growth, and agricultural advancements. It could also be evidenced by the rising number of publications in water treatment and desalination published over the last 30 years, as illustrated in Fig. 4a. Desalination methods are mainly classified into thermal and membrane-based technologies. According to prior publications (Fig. 4b), thermal and membrane procedures account for around 97% of the world's desalination capacity.^25^Fig. 5 describes the broad classification of desalination technologies.^26–29^ The principle of thermal-based technologies is to heat salt water to evaporate water vapour, which is subsequently condensed to produce fresh water.^30^ Mostly, all the large-scale thermal distillation facilities are found in the Middle East, especially in Saudi Arabia, where seawater serves as the primary supply of freshwater.^31,32^

**Fig. 6:**
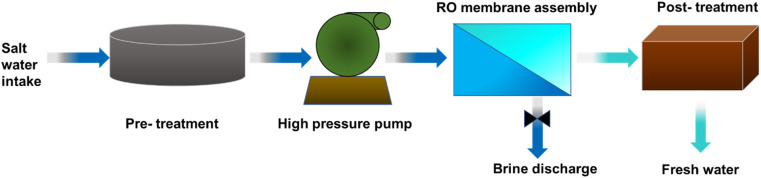
Flowchart for the RO process.

The invention of membrane-based technology signified a milestone in desalination since it can desalinate water with less specific energy consumption, a lower environmental impact, and a more flexible capacity. According to reports, membrane processes create 63.7% of the world's desalted water, demonstrating the significance of membrane technology in this application.^33,34^ Reverse osmosis (RO) employs a pressure gradient to transmit the high-pressure saltwater flow through a semi-permeable membrane to remove salt from water.^35^ High-pressure pumps are used in RO plants to exert this extra pressure, which forces saltwater across semi-permeable membranes to produce potable water. A flowchart for the RO process is shown in Fig. 6.^30,36–38^ The RO plant mainly consists of the following six fundamental components: (1) ingesting salt water, (2) pre-treatment facility to remove scaling and suspended particles, (3) high-pressure pump to confront osmotic pressure, (4) RO membrane with sufficient porosity to allow water molecules to flow through while preventing the entry of salt and other contaminants, (5) sustainable system for brine disposal, and (6) post-treatment system that includes pH adjustment, disinfection, and remineralization as necessary.^30^

Advanced technological developments have made water treatment more effective and affordable on a worldwide scale. This has been made feasible by innovations in materials, the utilization of renewable energy sources to lower the power requirement, and the integration of several treatment techniques into hybrid systems. In this review, we will highlight new directions for material advancement, employing IPN architecture for desalination and other water remediation.

## 4. Critical factors affecting advanced membrane technology and challenges

As previously mentioned, membrane technology has been the most widely employed among various other removal processes for purifying water. The membrane technology, however, is influenced by a variety of factors. RO-based membrane separation technology is the most widely used type, and it is effective at eliminating all salts and ions from contaminated water. Despite the impressive progress made by RO, more research is required to overcome existing challenges associated with RO operation, such as membrane fouling and scaling, which raises project costs, also insufficient removal of some trace-level dissolved substances, which may require additional treatment systems.^39^ Numerous innovative initiatives such as forward osmosis (FO), membrane distillation (MD), and membrane capacitive deionization (MCDI) have been reported in recent years that focus on strategies to create advanced polymeric membranes for membrane distillation (MD) that are less complicated, more affordable, and more environmentally friendly.^40^

### 4.1. Membrane fouling and scaling

Fouling is the progressive deposition of several unwanted elements on the membrane surface during its operation. The main types of foulants are includes particulate matter, organic compounds (such as polysaccharides, proteins, humic substances, nucleic acids, lipids, and amino acids), inorganic substances (which mostly cause scaling), and biological microorganisms (biofoulants).^41,42^ It has the effect of reducing membrane permeability, which is often countered by slightly increasing pressure to maintain the production of water. Over the life of the treatment plant, this drives up the cost of both power and water. Chlorine pre-treatment is used to reduce the growth of microorganisms and control biofouling. However, membrane lifespan is significantly decreased by exposure to chlorine.^40^ Further, different types of enzymes are used to minimize biofouling since proteins and polysaccharides are the two main structural components of biofilms. Protein-cleaving enzymes, such as trypsin and proteinase K, as well as polysaccharide-cleaving enzymes, such as alginate lyase, beta-mannosidase, and alginate lyase, were studied for their potential as anti-biofouling agents.^43,44^

Scaling is the development of a hard mineral layer as a result of the crystallisation or precipitation of soluble salts onto the membrane surface. Different types of scalants that develop on the membrane surface, including calcium carbonate (CaCO_3_), calcium sulphate (CaSO_4_), magnesium hydroxide (Mg(OH)_2_), barium sulphate (BaSO_4_), strontium sulphate (SrSO_4_), iron sulphide (FeS), and silicon dioxide (SiO_2_, silica).^45–47^ The desalination process can be inhibited or slowed down by mineral deposition on membrane surfaces at higher salt concentrations.^48^ Different antiscalant chemicals, such as polycarboxylates, which are naturally anionic low-molecular-weight polyelectrolytes, polyphosphates (such as sodium hexametaphosphate), phosphonates/organophosphates, and biobased antiscalants (such as carboxymethyl inulin), can be used to inhibit scaling.^45,49^

### 4.2. Energy recovery

Even though RO is the commercial desalination method with the highest energy efficiency compared to other thermal processes, it still uses a lot of energy. The RO process uses roughly 71% of the total amount of electricity used for desalination, while pre-treatment uses 11% and seawater collection, distribution, and ancillary facilities use the remaining energy.^48^ Additionally, there are growing initiatives to include renewable energy. There are several motivations for the use of renewable energy sources for water desalination, such as wind and solar, which is appealing due to the high cost of electricity. This is especially true for remote places where desalination is the only source of fresh water and energy is off-grid.^50^

### 4.3. Rejected brine

The management of concentrated rejected brine and its detrimental effects of it on the receiving environment are two key challenges in the desalination process. According to the literature, the majority of seawater RO plants overall can only recover between 30 and 50% of the feed water; if they recover more than this, the energy needed for separation rises exponentially.^40^ The rejected brine has a high concentration of salt, and additional chemicals from pre-treatment and cleaning processes, including iron chloride, sodium hypochlorite, and sodium bisulphite, which can have harmful impacts on marine life.^40,51^ It is challenging to accomplish zero liquid discharge (ZLD) and complete pure water recovery with RO alone. In a recent study, Panagopoulos *et al.* investigated several methods for achieving ZLD for the recovery of freshwater and solid salts using various cutting-edge technology.^52^

In recent years, research on desalination using polymer-based membranes has received a lot of interest. The desired characteristics in the polymeric membrane used for desalination include high liquid entry pressure (LEP), high permeability, tunable pore size, low fouling rate, low thermal conductivity, high thermal stability, and high mechanical strength. Cutting-edge research on membrane distillation has focused on modifying the properties of polymeric membranes to be suitable for the commercialization of this technology. Many novel efforts reported in recent years are focused on cheaper, simpler, and more environmentally friendly ways to fabricate polymeric membranes.^12,34,37,53,54^ These research efforts have contributed to a better understanding of the fabrication of high-quality polymeric membranes for desalination.

## 5. Plausible mechanism for IPN based water-remediating systems

In the design of water-remediating systems, an interpenetrating polymeric network can enhance and offer several advantages in pollutant removal mechanisms through membrane separation as well as adsorption. It prevents macroscopic phase separation in incompatible systems, enhances mechanical, chemical, and thermal stability, and helps to achieve tailorable surfaces and pores.^21^ Primarily, the contaminant removal *via* IPN-based membranes is driven by a host of combining factors including adsorption, repulsion, sieving, and complex formation. Intrinsic features such as pore size, wettability, surface charge, and operational conditions also have a profound impact on the rejection performance. Most of the rejection operations result from a synergism of size sieving and electrostatic interactions.^55–58^ More specifically, charge-bearing contaminants are either adsorbed or repelled by the charged membrane surface and pores. The Donnan exclusion principle plays a major role in the case of charge-based separation mechanisms.^59,60^ Additionally, to prevent fouling, the high chain densities of IPNs restrict the space between polymer chains, making it considerably smaller than the size of the foulant protein. Additionally, proteins may exhibit substantial steric repulsion toward polymer brushes with long graft chains, which might enhance the antifouling performance of the IPN-based membrane.^20^

In the case of size exclusion mechanisms, contaminants with a size greater than the pores get blocked whereas those smaller than the pores easily pass through. In the literature on membrane rejection for dye pollution, it is usually reported that membranes with pore sizes of 2–25 nm successfully reject the corresponding dyes and BSA.^61–63^ Specifically, the tunability of IPNs help create systems that can effectively tailor the pore size and the charge-carrying capacity of the systems, thereby augmenting final separation performance *via* the synergistic effects of pore-based and charge-based screening.^64^

**Fig. 7:**
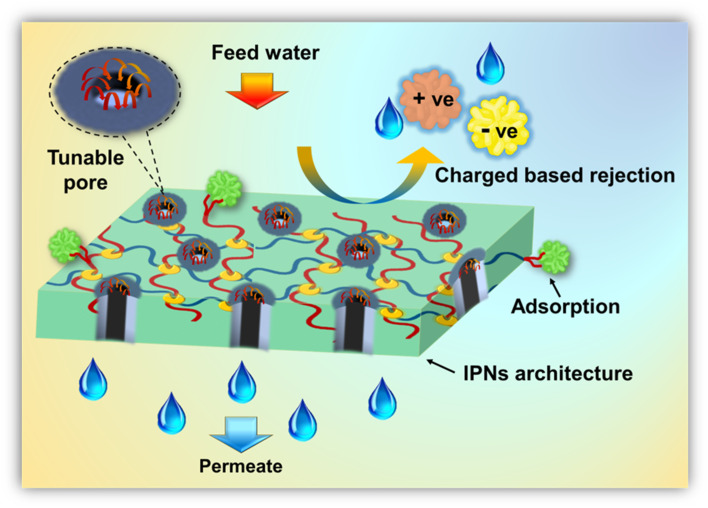
Plausible mechanism for IPN based water-remediating systems.

In systems other than membranes, such as adsorbents, including hydrogels, the primary mechanism of separations is facilitated by electrostatic adsorption, complexation formation between surface functional groups, and ion exchange reactions between adsorbent–adsorbate interfaces.^4^ However, the use of IPN strategies improves the modification of polymeric material structure, where the polymer develops from the primary polymer backbone by conventional polymerization.^20^ Another benefit of IPN is that different starting monomers may be used depending on the IPN type and contaminant, which improves pore wall and surface modification and create systems suitable for target-specific applications. The possible removal mechanism using IPN based structure is schematically illustrated in Fig. 7.

## 6. IPNs based membranes and adsorbents for water remediation

The IPNs have led to its utilization as a membrane, adsorbent, and hydrogel fabrication system due to their excellent mechanical, chemical, and heat resistance characteristics, as well as its variable pore size and shape. These membranes could be easily changed with doped conducting polymers for ion exchange applications to increase their hydrophilicity. Additionally, the polymeric functional group may readily bind with nanoparticles to make nano-enabled membranes and strengthen the substance to remove the targeted pollutant from water with greater effectiveness.^65^ The addition of different functional groups in the polymeric network might result in innovative performance for the substrate membrane.^66^

Polymer blends or mixtures make up the vast majority of the materials used in contemporary industry. However, this IPN structure represents one of the areas of polymer material engineering with the quickest growth. The multipolymer interpenetrating properties and methods of chemical and physical blending is advantageous in a variety of fields, as shown in Fig. 4c. IPN applications have risen gradually over the last several years, and significant research on water treatment and desalination has been published, as evidenced by the increasing publication Fig. 4d. In this review article, we will concentrate mostly on the application of IPN architecture for water remediation and desalination.

### 6.1. Desalination using IPN architecture

#### 6.1.1. IPNs based membrane for desalination

Over the last few decades, IPNs based membranes for desalination have gained great attention because of their high mechanical strength, good electrochemical stability, and compatibility. For instance, Sun *et al.* created microporous PVDF-polydimethylsiloxane(PDMS) semi-IPN membranes for vacuum membrane distillation by employing dibutyltin dilaurate (DBTDL) as a catalyst and tetraethylorthosilicate (TEOS) as a crosslinking agent. The structure and functionality of the produced membrane were investigated by varying the mass ratio of PDMS/TEOS. Membrane performance results reveal that NaCl salt (30 g L^−1^) rejection efficiency at a feed flow rate of 80 L h^−1^ is 99.9%, and excellent flux demonstrates the potential of semi-IPNs PDMS-PVDF membrane for seawater desalination.^67^ In a similar study, Qu *et al.* created semi-IPNs microporous PDMS/PVDF membranes by adjusting the mass ratio of PDMS/PVDF using a non-solvent-induced phase separation (NIPS) technique. The contact angle and mechanical properties were investigated in detail. The semi-IPN membranes demonstrated 99.9% desalting of NaCl solution (30 g L^−1^) and the performance was compared to that of the reported systems.^68^

**Fig. 8:**
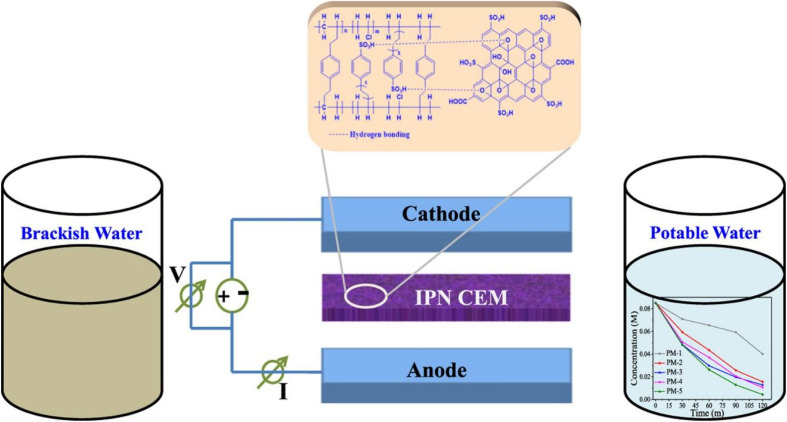
An illustration of the synthesis of SGO composite interpenetrating network (CIPN) cation exchange membranes (CEM) for the removal of salt (this figure has been reproduced from ref. 70 with permission from Elsevier, copyright 2018).

To improve the permselectivity and antifouling characteristic of conventional RO membrane, Wang *et al.* developed a Semi-IPN based on polyamide (PA) and poly (*N*-vinyl-2-pyrrolidone) (PVP). The modified membrane with optimal (0.5–10s-NVP) amount showed 43.2% and 0.2% increments in water flux and salt rejection, respectively. Furthermore, the antifouling performance was substantially improved; the 0.5–10s-NVP exhibited a lower decline ratio in the normalized water flux after the bovine serum albumin fouling process. This work provides a guideline for physicochemical properties and microstructural design of novel RO membranes.^69^ Rajput *et al.* synthesized interpenetrating network type composite cation exchange membranes (CEM) for water desalination. The membranes were prepared *via* solution casting method, using (polyvinyl chloride) PVC-St/DVB (divinyl benzene) with different content of sulfonated graphene oxide (SGO). An illustration of the synthesis of SGO composite interpenetrating network (CIPN) cation exchange membranes for the removal of salt is depicted in Fig. 8. Power consumption and current efficiency during salt removal were found to be significantly good (1.07 kW h kg^−1^ and 82%).^70^

A new polystyrene/PVDF cation exchange membrane with a semi-IPN structure is developed by Lei *et al.* at a reasonable cost but with comparatively great performance. The result shows that the sulfonation process may be affected by the cross-linked chain of IPN. The divinylbenzene (DVB) crosslinked polystyrene particles are so dense and hydrophobic that it is challenging for the hydrophilic sulfonation agent to penetrate the interior volume of the particles without the swelling agent (1,2-dichloroethane). The constructed membrane was successfully sulfonated to a degree over 80%. Additionally, a pilot-scale electrodialyser was used to assess the desalination effect and running electrical resistance of the produced cation exchange membrane, and it shows a satisfactory result.^71^

The Hydrophobic/hydrophilic interpenetrating network composite nanofibers (HH-IPN-CNF) for FO membrane support layer with high flux performance were fabricated by electrospinning approach by Tian *et al.* The membrane flux increased with the increase of PVA nanofiber content in the HH-IPN-CNF support layer. The optimized ratio of PET/PVA composite nanofibers resulted in high-water flux (47.2 LMH) and a low salt reverse flux (9.5 gMH), due to the existence of the HH-IPN-CNF structure which resulted in the improved wetting performance of support layer and the water-transferring function.^72^

In a recent study, Miao *et al.* created a unique modification approach that combines “Semi-IPN building” with “Photo-Fries rearrangement initiation”. Modified RO membranes with strong perm-selectivity and anti-fouling capabilities were created by adding acrylamide (AM), methacrylamide (MAM), and sodium allysulfonate (SA) to the aqueous phase, respectively, and applying UV irradiation during the interfacial polymerization (IP) process. The thin film composite reverse osmosis (TFC RO) membrane demonstrated excellent perm-selectivity and anti-fouling capabilities. The findings indicated that the water flux and NaCl rejection of the ideal modified membrane (MAM-UV) was 77.5 L m^−2^ h^−1^ and 99.44%, respectively, under the brackish water test conditions. Additionally, the anti-fouling test revealed that MAM-UV has effective anti-fouling characteristics against both bovine serum albumin (BSA) and lysozyme (LYZ). The innovative UV-initiated modification technique suggested in this study also offers a wide range of potential industrial applications.^73^

#### 6.1.2. IPNs based adsorbents for desalination

Recent studies have shown a variety of advanced IPNs architecture-based adsorbents, including hydrogel, that are used for desalination, as follows:

In a study, Xu *et al.* fabricated electro-responsive semi-IPN hydrogel composed of crosslinked poly(2-acrylamido-2-methyl-l-propanesulfonic acid-*co*-acrylamide) (P(AMPS-AM)) and linear polyelectrolyte polyacrylic acid (PAA). The porous structure of semi-IPN hydrogel was characterized by various techniques and was used for FO desalination application. The swelling capacity of the semi-IPN hydrogel, or the amount of liquid material it can retain, was 292.5 mg g^−1^ and when exposed to electric stimulus showed more pronounced deswelling behavior. From 2000 ppm NaCl solution, semi-IPN hydrogels showed 2.20 L m^−2^ h^−1^ (LMH). These results indicate that the proposed hydrogel has the potential for improving FO performance.^74^ Cai and his team designed thermally responsive semi-IPN hydrogels and were used as a draw agent in FO systems. The semi-IPN hydrogel showed good balance between thermally responsive swelling and dewatering behavior. The semi-IPN hydrogels released almost 100% of the water absorbed during the FO drawing process.^15^

Poly(acrylic acid-*co*-ethyleneglycol dimethacrylate) (XPAA) based IPN was prepared by Chanda *et al.* for the desalination of brackish water *via* thermos-reversible sorption process. The resulting IPNs, containing carboxylic acid groups and weakly basic tertiary amine groups in close proximity in the same resin bead, exhibited thermally regenerable desalination properties (*e.g.*, sorption of salt at 30 °C and desorption at higher temperatures (80 °C)), simulating the behavior of the well-known Sirotherm resins. The used resin was regenerated with 1 M NaCl solution at 90 °C and used repeatedly with no apparent loss of capacity, as demonstrated in the present work for up to 10 cycles of operation.^75^

**Fig. 9:**
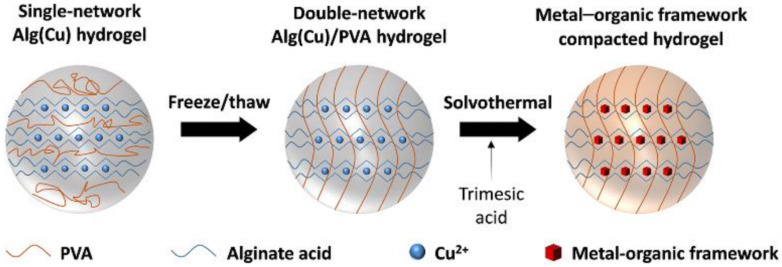
Schematic of the overall fabrication process of the proposed MOF incorporated MOF-Alg(Cu)/PVA hydrogel beads (this figure has been reproduced from ref. 76 with permission from Elsevier, copyright 2021).

According to Lee *et al.*, Cu-based alginate/PVA hydrogel (MOF-Alg(Cu)/PVA) beads were made *in situ* on an IPN using metal–organic frameworks (MOF). The developed MOF-Alg(Cu)/PVA beads demonstrate effective ion removal from artificial seawater and NaCl solution of various concentrations. Fig. 9 schematically demonstrated the overall fabrication process of the proposed MOF incorporated MOF-Alg(Cu)/PVA hydrogel beads.^76^

### 6.2. Dye removal using IPN architecture

There has been a global deterioration of the supply and quality of water and the availability of freshwater has emerged as the nexus of worldwide concerns. In recent years, various hazardous organic and inorganic compounds have been detected in wastewater, with dyes being a critical and significant class of harmful contaminants. Going by the statistics, it is estimated that annually nearly 7 × 10^5^ tons of dyes from the textile industries are being discharged into the industrial effluents.^77,78^ These organic dyes are highly toxic, pose carcinogenic and teratogenic possibilities and their traces in the water bodies can have detrimental effects on the ecosystem.^4,79^ Hence, it is the need of the hour to develop sustainable, yet affordable, robust, and eco-friendly techniques to remove these harmful and stringent contaminants from the wastewater streams. In this perspective, several physicochemical, as well as biological treatment methodologies, have been reported in the past to control and eventually eradicate such types of water pollution.

However, the usage of these techniques over a wide range of harsh operational conditions still poses as major roadblocks towards their practical scalability. With this motivation in place, researchers across the globe have been in continuous pursuit of techniques that are robust, tuneable yet affordable for the general mass. To this end, the deployment of IPNs for stable removal of organic dyes from wastewater streams can offer innumerable advantages. They help in realizing systems that can achieve significantly improved targeted separation performance owing to their inherent characteristics.^64,80–83^

#### 6.2.1. IPNs based membranes for dye removal

IPN-based membrane synthesis and design have been investigated by many researchers for the removal of dye from polluted water, due to its numerous benefits, including its ability to customize physical properties like pore volume, size, and surface area. IPNs also make it feasible to enhance the membranes' chemical and mechanical resilience by avoiding macroscopic phase separation. IPNs architecture could also be applied to modify the chemical composition of selective layers, which will significantly enhance the separation performance of the final membrane for removing dye contaminants from water.^20,64^

**Fig. 10:**
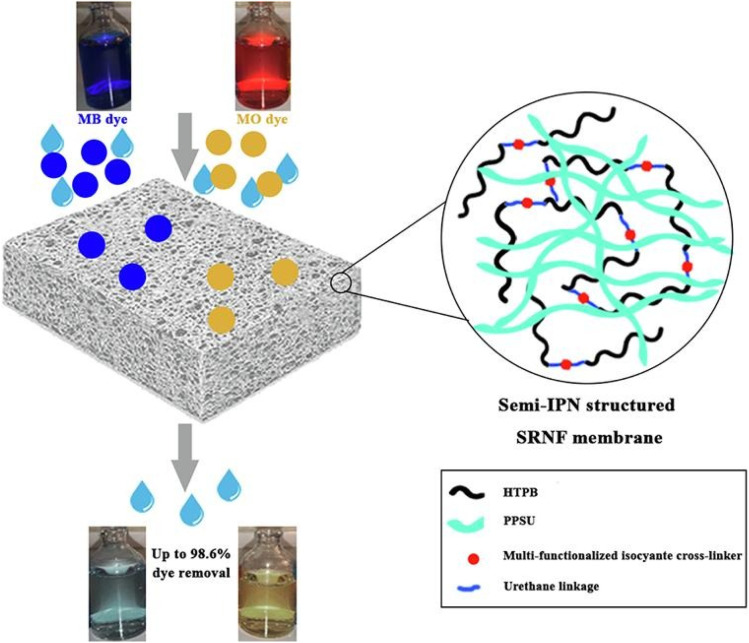
Schematic representation of the structure of semi-IPN based solvent resistant nano-filtration (SRNF) membrane and its MB and MO dye removal performance (this figure has been reproduced from ref. 83 with permission from Elsevier, copyright 2022).

Nozad and his team created a semi-IPN type membranes by incorporating HTPB (Hydroxyl-terminated Polybutadiene) and MFI (Multifunctional Isocyanate) into a polyphenylsulfone membrane matrix. The membranes were highly chemically resistant to solvents like DMF (*N*,*N*-dimethylformamide) and could successfully remove 98% of both cationic MB and anionic methyl orange (MO) dyes and Fig. 10 provides a schematic representation of a general overview of the procedure.^83^

Sen Gupta *et al.* reported a novel yet facile pH-responsive mussel inspired sequential-IPN membrane with PVDF (polyvinylidene fluoride) and dopamine monomers. With the aid of charge as well as pore-based screening, the membranes were made selective towards particular dyes and stringent antibiotics. Over repeated operational cycles, the membranes eliminated >97% of both anionic and cationic dyes in acidic, basic, and neutral conditions. The membranes also removed charged pharmaceutical compounds and exhibited cytocompatibility with respect to mammalian cell lines.^64^

**Fig. 11:**
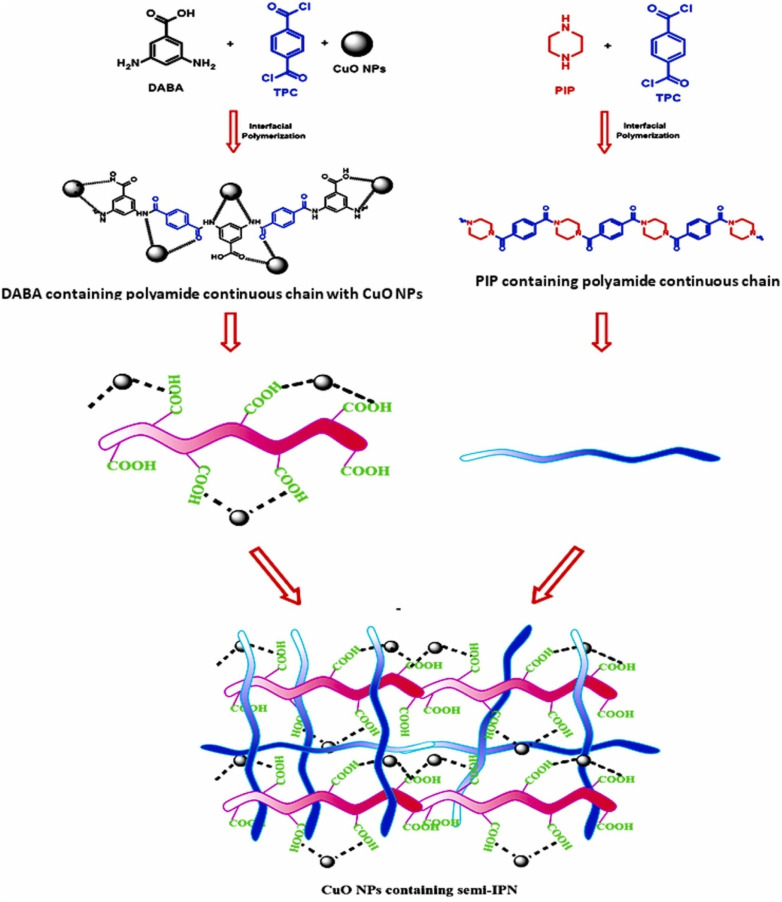
Schematical illustration of reaction conditions and proposed structures of the polyamide chains and semi-IPN (this figure has been reproduced from ref. 85 with permission from Elsevier, copyright 2022).

Shi *et al.* developed a porous interpenetrated membrane by deploying Chitosan and hydroxyapatite. In less than 15 min the membranes removed 98% of DB (direct black) dye and retained 80% of such efficiency after 5 cycles of dynamic operations. The feed concentrations used here was as high as 150 ppm.^84^ Waheed and his team developed a novel TFC (thin film composite) membrane with semi-IPN architecture by using PIP (piperazine) and DABA (3,5-diaminobenzoic acid). They subsequently immobilized CuO onto the active layer. In Fig. 11, they have schematically illustrated the reaction conditions, suggested structure of the polyamide chains, and the resultant semi-IPN. Although the membrane allowed easy permeation of several salts they successfully rejected >99% of MB dye.^85^

Quaternized PVA and acrylamide were used for the fabrication of alkaline full-IPN membrane and it involved the *in situ* polymerization of Gemini cationic molecule. This system was developed by Wang and his team to investigate the adsorptive removal of RB and CR dyes in single and binary systems. In binary systems, a synergism arised due to the interaction between CR and RB dyes. Results indicated that the amount of CR removal is higher than RB in binary and single systems.^86^

In another work, Zhai *et al.* successfully synthesized double-layered membranes with a semi-IPN top layer and a catalytic 2nd layer. It was a PVDF/Pd composite membrane that could catalytically degrade *p*-nitrophenol and could remove >99% of CR, direct black 38, direct red 23 dyes, and BSA pollutants.^63^

#### 6.2.2. IPNs based adsorbents for dye removal

In a study, Mandal *et al.* fabricated semi-IPN and IPNs with PVA (polyvinyl alcohol) and copolymer derived from AA (acrylic acid) and HEMA (hydroxyethyl methacrylate), *i.e.* poly(AA-*co*-HEMA). They study their adsorption capacity from low concentrations of aqueous solutions *via* using cationic dyes RB (Rhodamine B) and MV (Methyl violet) as the model dye foulants. At pH 7 with increase in copolymer content, the sorption capacity for both dyes were found to increase. Simultaneously it was also observed that the MV sorption was invariably higher than that of RB, owing to the positive charges of RB as well as the presence of carboxylic groups which ionized (at pH 7) and repelled similar groups of the IPN. Additionally, the tighter network structure of the IPNs accounted for better adsorption and removal of the dyes as compared to the semi-IPNs.^87^ Ray *et al.* investigated the effect of dye concentrations present in the feed on the mechanism of binding cationic dye foulants (here MV and basic Fuchsin) onto the semi-IPNs which they synthesized using CMC(carboxymethyl cellulose) and copolymer of AM (acrylamide) and HEMA. Their findings suggested that all the semi-IPNs exhibited a shift of chemical to physical sorption on moving from low to high dye concentrations.^88^ Şolpan *et al.* reported the fabrication of semi-IPN hydrogels from SA (sodium alginate) and PAAm (polyacrylamide), wherein 3 wt% of the alginate was incorporated into the PAAm matrix. Subsequently, they were used for removing textile dyes. The hydrogels followed the order of MV > methylene blue(MB)>Safranine-O > Magenta for the adsorption capacities at a pH of 7 and a temperature of 25 °C.^89^ A crosslinked network structure was obtained by Sharma *et al.* from grafting of itaconic acid (microwave initiated) on guaran polysaccharide. A dual step aqueous state polymerization was adopted for obtaining conducting IPN, where, aniline was absorbed into the semi-IPN framework. The conducting IPN removed 84.5% and 81% of MB dye under neutral and acidic environments respectively.^90^

A novel biodegradable IPN was reported by Saruchi *et al.* from Av (aloe vera) and AA with APS (ammonium persulfate) as the initiator and MBA (*N*,*N*′-methylene bisacrylamide) as the crosslinker. A microwave-assisted technique was used to synthesize the IPNs. The biodegradability of the IPN were studied using composting and soil burial methods. With the composting method, 94% degradation (70 days) was obtained from composting technique and 86% degradation (77 days) was reported from the soil burial method. The IPNs removed 94% of cationic Malachite green (MG) dye and the adsorption procedure was found to correlate with the pseudo-second-order model. Langmuir isotherm was found to be the best suited and fitted isotherm model.^91^ In a similar work by the same group, similar IPNs were fabricated, however this time without the aid of microwave-assisted technique.^80^ The alteration in the fabrication technique was found to result in 97.3% removal of MG dye.

In a recent work by Hosseini *et al.* a semi-IPN type anionic hydrogel was reported which was synthesized from XG (xanthan gum), GO (graphene oxide), and cross-linked polyacrylic acid. This superabsorbent was essentially meant for MB removal from wastewater. A synthetic unique crosslinker based on acrylic-urethane (MS) was deployed to crosslink AA in XG and GO solutions. The hydrogels exhibited pH-responsive behavior with respect to water uptake and demonstrated a spectrum of swelling ratios with the highest being in alkaline media and the lowest in acidic media.^92^ The nanocomposite adsorbed 88.5% of MB dye and swelled up to 485%. Various models such as Elovich kinetics, 1st order, and intra-particle diffusion models were in well agreement with the experimental findings.^92^ In another work, smart filters based on full-IPNs were synthesized by Elella and team with XG and TMC (*N*,*N*,*N*-trimethyl chitosan). The filters were able to remove nearly 94.4% of cationic crystal violet (CV) dye and an inhibitor for the growth of *Escherichia coli* from wastewater.^93^ In a completely new direction, Sharma *et al.* synthesized Guar gum (a polysaccharide) based hydrogels attributed with both biodegradability and conductivity. Apart from guar gum, acrylic acid was used as the second component in the hydrogel system along with APS (ammonium persulfate) and hexamine as the initiator and crosslinker respectively. The crosslinked semi-IPN hydrogels were doped with HCl which in turn accounted for their conductive characteristics. With these characteristics, the hydrogels were used for antibacterial studies and dye adsorption efficiencies. The samples exhibited the presence of inhibition zones for *E. coli* and *S. aureus* bacterial cell lines. Variations of several conditions such as adsorbent dosage, pH, temperature, *etc* were found to profoundly affect the MB dye adsorption efficiency. Nearly 94% of dye adsorption was obtained by varying the temperatures.^94^ In another gum-based work, guar gum, KHA (potassium humate), and PVA were employed to fabricate a degradable IPN hydrogel by Niu and his group. The study result demonstrated that the maximum adsorption capacity of the prepared hydrogel is 1166.73 mg g^−1^ and 625.21 mg g^−1^ for MB dye and Pb(ii), respectively. While analyzing the thermodynamics of the process, it was revealed that the adsorption of MB and Pb(ii) were intrinsically endothermic in nature and the process was spontaneous and it was driven by an increment of entropy.^95^ In a similar gum-based work by Kumar *et al.*, KG (katira gum) and PVA were used for making microspherical semi-IPNs. The crosslinker used for the synthesis was glutaraldehyde. The microspheres were effective in removing nearly 96.92% of Bismark brown-yellow dye at a feed concentration of 5 ppm. Spontaneity of the process was suggested from thermodynamic data (Δ*G*° = −1.9, 3.5, & 4.0 KJ mol^−1^) and the evident endothermic nature (Δ*H*^0^ = 29.22 KJ mol^−1^) of adsorption at specific temperatures were found to exhibit positive entropy (Δ*S*^0^ = 103.53 J mol^−1^).^96^

In another work, Taktak *et al.* synthesized XG-cl-2-(*N*-morpholinoethyl methacrylate) (MEMA)/titanium dioxide (TiO_2_) semi-IPN hydrogel composites and used them for single and binary removal of cationic dyes. The hydrogels showcased an adsorption capacity of 63.34 mg g^−1^ and 83.25 mg g^−1^ of MB and CV dyes respectively.^97^ Further, Singha and his team fabricated a sustainable yet biofriendly guar gum-*g*-(acrylic acid-*co*-acrylamide-*co*-3-acrylamido propanoic acid) hydrogel and used it for the removal of several heavy metals and toxic dyes. 27.06 mg g^−1^ and 39.35 mg g^−1^ were the maximum adsorption capacities reported for MB and SF (safranine F) dyes respectively.^98^ Sukriti *et al.* reported the synthesis of a semi-IPN hydrogel from XG, PVA, and tartaric acid. With the usage of 700 mg of adsorbent dosage, the material could effectively remove 70% and 63% of cationic dyes such as RB and AO (auramine-O) respectively.^99^ In a unique approach, Kaur *et al.* synthesized an IPN hydrogel with collagen and reduced gum copal resin, ((GcA-coll)-cl-poly(AAm-ip-AA)) and the copolymerization was carried out with acrylamide chains and acrylic acid as the rightful 2nd monomer. The hydrogels were used for the removal of MB dye from aqueous streams. At pH 7, the maximum adsorption capacity was recorded to be 1.7 mg g^−1^ with an adsorbent dosage of 300 mg, and repeated regenerations were successfully possible with this material.^100^ In another similar work by the same group, similar microwave-induced IPN gum-based hydrogels were synthesized for the effective removal of cationic dye MG dye from wastewater. The percent swelling of the synthesized systems was gauged and optimized from the RSM (response surface methodology). Nearly 88% of dye removal was achieved at 7 pH with 500 mg of adsorbent dosage.^101^ Another group, Sharma *et al.* were actively involved in the fabrication of semi-IPN based superabsorbent resulting from gum acacia and sodium alginate. A maximum swelling capacity of 1729.4% was achieved for these adsorbents and could selectively remove nearly 95.39% of MG dye, 94.56% of CV, and 97.49% of AO (auramine-O). Adsorption efficiencies for AO, CV, and MG were recorded at 2.01 mg g^−1^, 7.55 mg g^−1^, and 3.06 mg g^−1^, respectively. The desorption of the dyes were easily carried out *via* 0.1 N HCl.^102^ In another study, Sharma *et al.* synthesized nano spikes based on semi-IPNs of poly(acrylamide-aniline)-grafted gum ghatti. The system was targeted towards the adsorptive removal of MG dye and a maximum of 98% removal was achieved after 20 h of usage.^103^

In a completely different work, Anirudhan and his team developed an IPN-based hybrid hydrogel and used it for studying the removal efficiency of MB dye. Graft copolymerization technique was used to initiate the reaction amongst Cell (cellulose), Bent (bentonite), and PMAA (polymethacrylic acid). K_2_S_2_O_8_ (potassium peroxide) and MBA (*N*,*N*′-methylenebisacrylamide) were used as the initiator and crosslinker respectively. At an optimal pH value of 6.5, in a batch process, 99.9% of MB adsorption was recorded. Pseudo-second order equation was found to be best suited for describing the adsorption process. SIPS adsorption-type isotherm could well explain the equilibrium data for the MB dye adsorption. Desorption studies indicating the reusability of the hybrid hydrogel demonstrate its potential use for sustainable and affordable usage.^14^ In a new disposition, Aljar and group focussed on developing a semi-IPN type nanocomposite hydrogel for efficient removal of cationic MB dye from water streams. The hydrogel consisted beads of PVA (polyvinyl alcohol) and Alg (alginate) and was impregnated with Bent (natural bentonite) which would play the role of a beneficial adsorbent. The Bent concentrations in the porous beads were varied and the synthesis was realized using ionic gelation technique. As compared to the beads without Bent, the ones with 30 wt% of Bent demonstrated a colossal improvement in the removal efficacy (by *c.a.* 230%). Even after six repetitive cycles of adsorption–desorption, the beads were found to retain nearly 90% of their efficiency.^104^

Semi-IPNs composed of AA, acrylamide, and PVA were reported by Zendehel *et al.* and they too aimed at the removal of MB dye from aqueous systems. Although the composites could remove 95% of the azo dye, desorption studies with these hydrogels did not yield any satisfactory results. These materials can thus be deployed as membranes for efficient dye removal instead of being used as adsorbents.^105^ Sarkar *et al.* reported the fabrication of affordable and low-cost polyacrylamide hydrogel induced with super porosity. As a reinforcing agent, they incorporated nano-CaCO_3_ into GO. The resultant nanocomposite hydrogel was effective in removing nearly 98% and 97.6% of cationic MB and RB dye, respectively. The performance retention was well even beyond 5 cycles of desorption and adsorption. The removal efficiency of anionic MO dye was retained at 94.45% even after similar cycles of repeated operation.^106^

A stimuli-responsive, more specifically pH-responsive superabsorbent based on PVA, PAA, and yeast was fabricated by Feng *et al.* and the fabrication was realized using solution polymerization technique. From the measurements of mechanical integrity, it was concluded that the adsorbents could easily resist up to 5000 rpm of shear flow, and withstand 3 kg of load pressure. Just like the previous study, here too several parameters were varied to study the adsorption performance of the materials. The maximum adsorption capacity was recorded at 50 mg g^−1^ for MB. The adsorbents also exhibited several cycles of repeated adsorption and desorption, which hinted at their facile reusability and regeneration.^107^ Wu *et al.* synthesized a variety of semi-IPN polysaccharide hydrogels from PDA, pullulan and used HDE (1,6-Hexanediol diglycidyl ether) as the crosslinker. The hydrogels exhibited 107 mg g^−1^ adsorption of cationic CV dye and even after 4 repeated cycles of operation, the adsorption efficiency was retained at 100 mg g^−1^. The hydrogels were found to follow Langmuir and pseudo-second-order models. Being generated from non-toxic and cheap raw materials, the hydrogels have the potential to be upscaled industrially for sustainable yet rapid dye removal applications.^108^ A unique semi-IPN hydrogel-based composite was fabricated by Rahmatpour *et al. via* embedding PVA and GO nanosheets within partially hydrolysed and crosslinked networks of PHPAm (polyacrylamide). The preparation was done in aqueous solution *via* adopting an ionic internal gelation technique. The basic incorporation of GO into the IPN system resulted in enhanced swelling ability, superior thermal stability and increased the MB removal efficiency by nearly 50%. Even after continuous adsorption and desorption cycles after 5 times, 70% removal efficiency was retained. The maximum adsorption efficiency (as obtained from the Langmuir isotherm model) was found out to be 714.8 mg g^−1^ at 30 °C.^82^

Junlapong *et al.* incorporated CS (cassava starch) and PAM (polyacrylamide) in the preparation of a superabsorbent hydrogel by radical polymerization. The hydrogel with 50 wt% of CS absorbed >8000% of water and was able to remove >85% of MB within less than 10 h. The maximum recorded absorption capacity was 2000 mg g^−1^. The results were found to fit best with the pseudo-second-order and Freundlich model. Even after 4 repeated cycles of operation, the removal efficiency was retained at >50%. These hydrogels were found to exhibit both photodegradation and biodegradation behavior which would further add on to their sustainable usage.^109^ Maity *et al.* synthesized both semi as well as full-IPN based hydrogels from chitosan and methacrylic acid by using crosslinkers MBA (*N*,*N*′-methylene-bis-acrylamide) for semi and glutaraldehyde for full-IPN. The initiator and crosslinker concentrations were varied for the synthesis of several such systems. These systems were used for studying the removal of cationic methyl violet (MV) and anionic Congo red (CR) dyes. Studies indicated that the synthesized systems could effectively remove 91% of MV and 82% of CR model dye foulants.^81^

An interpenetrated hydrogel porous structure was developed by Maijan *et al.* using PVA and PAM (polyvinyl alcohol-*g*-polyacrylamide). Within this hydrogel, ZnO nanoparticles modified with SiO_2_ layer was introduced. This step facilitated the formation of silane bridges within the hydrogel. Nearly 8000% water absorbency and 96% removal of MB dye within a time span of 24 h was recorded for this system. A maximum absorption efficiency was recorded at 757 mg g^−1^. UV exposure was able to decompose the adsorbed MB dye at a rate of 0.1019 h^−1^. The surface coating of SiO_2_@ZnO also aided the degradation behavior. This hydrogel has the potential to be applied for circular economy and thus effectively remediate wastewater streams.^110^ Sadik and his team reported a facile and fast synthetic procedure for making a hydrogel based on starch-grafted poly(*N*,*N*-dimethyl acrylamide) *via* microwave-assisted technique. A maximum of 91% removal of Acid Red 8 dye was obtained at an optimum pH of 1. An adsorption capacity of 120.48 mg g^−1^ was obtained and the studies fitted well with Langmuir isotherm model.^111^

Zhao and his group prepared composites based on semi-IPN hydrogels *via* copolymerization of PEG (polyethylene glycol) and Aam (acrylamide) monomer within which CS (chitosan) was embedded. They were for investing the swelling properties and the kinetics of the hydrogels in aqueous solution and in the presence of AR 18 (Acid Red) solution. These hydrogels were better suited for the adsorption of anionic dyes (AR 18: 342.54 mg g^−1^, acid orange 7: 221.1 mg g^−1^ and MO: 185.24 mg g^−1^) as compared to the cationic ones (basic violet 14). Langmuir equation was found to suit the adsorption process and the mechanism behind the enhanced adsorption can be attributed to the electrostatic interaction between the negatively charged anionic dyes and the functional groups present in CS.^112^

A series of semi as well as full-IPNs based on PAAm and CS hydrogels were prepared by Dragan *et al.* The optimization of the fabrication process was based on several factors such as pH of the reaction mixture, the crosslinker ratio, and, the molar mass of CS used. The semi-IPNs had only positively charged functional groups and thus adsorbed a high amount of anionic dye (direct blue 1: 2.804 mg g^−1^) as compared to the full-IPNs (0.396 mg g^−1^). The exact contradictory behavior was observed in case of cationic MB dye where the full-IPNs (6.744 mg g^−1^) absorbed more as compared to the semi-IPNs (0.392 mg g^−1^).^113^

In another work by the same group, a semi-IPN hydrogel was synthesized composed of polyacrylamide (PAAm) matrix and potato starch (PS) or the hydrolyzed PS-*g*-PAN. The adsorption of MB dye was studied with this material. Nearly 6 mg g^−1^ of dye was adsorbed by this material.^114^ In similar lines, working with IPN membranes, Hu *et al.* made a full-IPN gel with Ca^2+^ crosslinked alginate network and integrated a covalently crosslinked poly(acrylamide) network. Subsequently, they hybridised PEG (polyethylene glycol) and POSS (polyhedral oligomeric silsesquioxane) for better performance. With 500 ppm of dye feed concentrations, they were able to reject 93% of MB and 95% of CR dye.^115^ Wang and his group synthesized semi-IPN hydrogels from CS and PAA with MBA as the crosslinker. Within 45 minutes of contact adsorption equilibrium was achieved. At a pH of 10, the hydrogels adsorbed a maximum of 430 mg g^−1^ of basic BB12 (Nil Blue) dye. The adsorption studies could be well ascribed to the 2nd-order kinetics *via* Langmuir isotherm.^116^ Al-Mubaddel *et al.* prepared chitosan (CS)/polyacrylonitrile (PAN) blend and semi-interpenetrating polymer network (sIPN) hydrogel films for the adsorption of Rhodamine B dye. The adsorption kinetics of Rhodamine B (RB) was observed to be pseudo-second-order equation and the equilibrium adsorption obeyed the Langmuir isotherm equation. Besides, kinetics and adsorption isotherm study revealed that the absorption mechanism is chemical in nature.^117^

For the first time, Abebe *et al.* synthesized Methylcellulose/tannic acid complexes which were coated on alginate IPN scaffolds for the removal of MB from waster medium and QUI (quinoline) from non-aqueous medium. 791.17 mg g^−1^ MB and a high amount of 460.92 mg g^−1^ QUI was adsorbed *via* these systems.^118^ Kalkan *et al.* synthesized a series of semi-IPNs based on poly(*N*-isopropylacrylamide-*co*-methacrylic acid)/poly(acrylamide)/SiO_2_ gels. The material was found to remove nearly 75% of methyl violet (MV) dye.^119^ Novel cellulose acetate/acrylic acid-glutaraldehyde semi-IPNs were synthesized by Rana and his team and the designed materials were meant for targeting the removal of MB dye from wastewater. The synthesized systems were found to remove >90% of MB dye within short intervals of contact time.^120^ A unique solid-state liquid crystal shell IPN hydrogel was prepared by Gwon *et al.* The IPN systems could successfully reject nearly 99% of MB and Acid Red 37 dyes.^121^

Toledo and his group could synthesize a novel carboxymethylcellulose and polyacrylic acid-based IPN hydrogels and deploy them for multifaceted adsorption applications. Cu(ii) ions and MB dye were the main focus of their removal studies. The adsorption maxima for Cu(ii) and MB dye was found to be 250 mg g^−1^ and 613 mg g^−1^ respectively.^122^ Bai and team developed IPNs with PVA and cellulose nanocrystals by photo crosslinking and freeze-thaw process. *Via* several modifications, a wide range of such hydrogels was formulated. The hydrogels showcased tremendous adsorption against toxic dyes such as MB and XO (xylenol orange). The maximum removal efficiency was recorded at 91% for MB and 93% for XO model dye foulants.^123^

A pH and temperature sensitive semi-IPN structure cellulose filament (CF)-poly (*N*-isopropylacrylamide-*co*-acrylic acid) hybrid hydrogel was fabricated by Zhang *et al.* and the resultant adsorbent was used for the adsorption of MV dye. The hydrogel was found to successfully remove 226.02 mg g^−1^ of the dye pollutant.^124^ Ngwabebhoh and the group reported chitosan and starch-based IPN hydrogel for the effective removal of DR 80 (Direct Red) dye. The hydrogel could swell maximum until 15 mg g^−1^ and the adsorption efficiency for the dye was found to be 312.77 mg g^−1^.^125^ A novel TiO_2_ nanoparticle immobilized SA (sodium alginate) and MAA (polymethacrylic acid) based hydrogel adsorbent was synthesized by Škorić *et al.* and the nanocomposite was utilized for the adsorptive removal of MB dye from wastewater. The resultant hydrogels were highly efficient and were found to remove nearly 93% of the toxic azo dye.^126^

In another work, ALSamman and team designed chitosan and alginate-based hydrogels with semi-IPN structure and deployed them for dye removal properties. The hydrogels were found to remove almost 96% of MB dye within 10 minutes.^127^ Properties such as compression-based elasticity and varied stimulus response were studied using semi-IPN hydrogels from acylamide and itaconic acid in the presence of various concentrations of the natural polymer gelatin (GLN) by Ciftbudak *et al.* The synthesized materials also demonstrated promising dye adsorption properties. Nearly 15 mg g^−1^ of MB and 8 mg g^−1^ of MG dye foulants were adsorbed *via* these systems.^128^ Cui and team developed porous hydrogel materials based on chitosan and gelatin and crosslinked them using genipin. The porous composites were effective in removing nearly 550 mg g^−1^ of AO II (acid orange) dye foulant.^129^

Wang *et al.* fabricated an *in situ* crosslinked PES (polyethersulfone) and modified chitosan-based hydrogel adsorbent. The hydrogels were deployed for the removal of harmful phenols and cationic CR dye from wastewater systems. Nearly 80% of dye removal was achieved apart from the removal of other heavy metals and phenols.^130^ Zhang *et al.* were responsible for the synthesis of hPEA (hyperbranched Poly(ether amine))/PVA IPN. The synthesis was realized *via* aldol condensation reaction. The fabricated composites were able to effectively remove nearly 90% of several fluorescein dyes.^131^

A diverse-stimuli magnetically doped hydrogel was reported by Ahmad and his team and the synthesis was based upon PNIPAM (poly(*N*-isopropylacrylamide)) and PMAA (poly(methacrylic acid)). About 3.5 mg g^−1^ of Magenta dye was absorbed apart from 0.7 mg g^−1^ of CR dye.^132^

In the aforementioned studies, some relevant works have been highlighted which clearly demonstrate the usability and establish the relevance of IPNs in the field of dye adsorption and separation. However, a major bottleneck arises from the limited reusability of these materials over several operational cycles. Efforts need to be directed to overcome such situations and come up with IPN-based solutions that are sturdy, affordable, and yet sustainable for long-term usage.

### 6.3. Miscellaneous water remediation application using IPNs based material

In addition to uses for desalination and dye removal, IPN is also applied to remove other water contaminants, including heavy metals, antibacterial agents, and microbial activity, some of which are listed in the literature below:

Wang *et al.* and team developed a bottom poly(vinylidene fluoride)@Pd catalytic layer and a semi-IPN coating layer. The pollutant Bovine serum albumin (BSA) is immediately separated by the membrane in a continuous cross-flow filtering process, whereas the pollutant *p*-nitrophenol (PNP) enters through the semi-IPN top layer and subsequently reacts in the catalytic layer. The final product obtained is *p*-aminophenol (PAP), which is later separated and collected *in situ*. Additionally, they noted that the produced membranes have molecular weights of about 500, 493, and 484 g mol^−1^, significantly less than the 68 000 g mol^−1^ of BSA. As a result, the smaller pore size of the bilayer membrane effectively rejects the BSA.^133^

Wang and his colleagues fabricated a cost-effective chitosan/gelatin-based IPN sponge incorporated with melanin-coated titania hollow nanospheres (CG@MPT-h) for solar-driven wastewater treatment application. The embedding of MPT-h nanoparticles in the IPN sponge effectively inhibits the growth of bacteria in the vertical channels, resulting in an antibacterial solar-driven water evaporator.^134^

Wang *et al.* developed a variety of super-absorbent semi-IPN hydrogels using a free radical polymerization process with ultrasound assistance. Further, the adsorption of cobalt(ii) from aqueous solutions was examined, with the removal effectiveness being greatest at pH 4. Adsorption kinetics and isotherms for cobalt(ii) were determined to be in accordance with the pseudo-second-order model and the Freundlich model, respectively. Additionally, thermodynamic parameters for cobalt(ii) adsorption were computed, and the results indicated that the adsorption behavior is spontaneous and endothermic.^135^ Zhao *et al.* effectively combined polyacrylamide (PAM) cross-linked by *N*,*N*-methylenebis (acrylamide) with α-ketoglutaric acid grafting chitosan (KCTS) to develop a semi-IPN hydrogel for the removal of heavy metal ions. The maximum adsorption capacity of Cu(ii), Pb(ii), and Zn(ii) by KCTS/PAM hydrogel at 30 °C and pH 5.0 were 72.39, 61.41, and 51.89 mg g^−1^, respectively. The produced hydrogel has a porous structure with a large specific surface area, and it exhibits a reusability property for successfully removing heavy metals.^136^

Chen *et al.* created a brand-new nitrogen-doped carbon dots (NCDs) crosslinked, cellulose nanofibril (CNF) and chitosan (CS) based IPN hydrogel (NCDs-CNF/CSgel) for the simultaneous fluorescence detection and adsorption of Cu(ii) cation and Cr(vi) anion. The results demonstrated high adsorption capabilities of 148.30 mg g^−1^ for Cu(ii) and 294.46 mg g^−1^ for Cr(vi), respectively.^137^

To remove heavy metals, Tanan *et al.* prepared IPN hydrogels of poly(2-hydroxyethyl methacrylate-*co*-acrylamide)/poly(vinyl alcohol), (P(HEMA-*co*-AM)/PVA) employing microwave-assisted (IPN-MW) and conventional thermal heated (IPN-TH) techniques. IPN-MW adsorbent had a maximum Pb(ii) adsorption capacity of 292.5 mg g^−1^, which was 4.51 times more than IPN-TH hydrogel's (64.8 mg g^−1^).^138^ Al-Sakkari *et al.* were involved in testing and fabricating new IPNs based on carrageenan, bentonite, and sodium alginate. They were aimed at removing heavy metals and toxic dyes from water bodies. The IPNs were found to report an adsorption capacity of 1500, 1550, 1540, and 1271 mg g^−1^ for Ni^2+^, Fe^3+^, Cr^3+^, and MB dyes respectively. 5 repetitive regeneration cycles were possible with this IPN formulation and this was also deployed for real-time contaminated water streams from oasis groundwater and tannery plant.^139^

In a different study, Wang *et al.* created an IPN gel by polymerizing acrylamide (AAm) and 1,4-butanediol vinyl ether(BVE) using a free radical/cationic hybrid process. These gels were used to separate the ions Cu^2+^, Ni^2+^, and Zn^2+^ in samples of natural water.^140^

In related work, Wang and his team created hydrogels with improved adsorption capabilities for the removal of heavy metal ions made of poly(polyethylene glycol diacrylate), poly(PEGDA), and poly(methacrylic acid) (PMAA).^141^ In another work, Hu *et al.* also designed hybrid IPNs based on copolymer of poly(di(ethylene glycol)) and methyl ether methacrylate in alginate–Ca^2+^ hydrogels containing the photocatalyst graphitic carbon nitride (g-C_3_N_4_). The designed hybrid hydrogels could successfully remove 96% of the MB dye from wastewater while having high recyclability.^142^

In order to produce an IPN, Peñaranda *et al.* used novel starch/acryl amide-based hydrogels in the presence of lignin or peat to remove Cu^2+^ and Ni^2+^ from polluted simulated water.^143^

The sodium alginate (NaAlg)/acrylamide (AAm) IPN was created by Şolpan *et al.* at three distinct compositions, with the sodium alginate content varying by 1, 2, and 3% (w/v) in solutions containing 50% (w/v) acrylamide. The produced material demonstrates a successful removal of the heavy metals Ni^2+^, Cd^2+^, and Pb^2+^.^144^ Soyekwo *et al.* were fabricated an IPN by crosslinking PVA and integrating Zn^2+^ moieties *via* coordination with the amino groups of crosslinked polyethyleneimine (PEI). To create the composite membrane, an intermediary layer of borate crosslinked polydopamine-grafted carbon nanotubes (B-PDA-CNT) was then added to the microfiltration substrate. The study showed that positively charged membranes in acidic environments provide strong rejection (>85%) of heavy metal ions such as Cd^2+^, Cu^2+^, Zn^2+^, Pb^2+^, and Ni^2+^. The further membrane is appealing for recovering phosphorus under low pressure.^145^

In order to improve the tensile strength, water swelling, and antibacterial activity, Panpinit *et al.* developed a novel poly(2-hydroxyethyl methacrylate-*co*-acrylamide)/polyvinyl alcohol/chitosan (P(HEMA-*co*-AM)/PVA/CS) IPN-CS hydrogel films *via* two-step free radical polymerization. The IPN-5%CS hydrogel film had a tensile strength of 22.4 MPa, which was 2.7 times more than that of IPN-0%CS. Using the viable cell counting method, *Escherichia coli* (*E. coli*, ATCC25922), a Gram-negative bacterium frequently seen on burn wounds, was used to assess the antibacterial activity of all IPN-CS hydrogels. According to these findings, IPN-CS hydrogel films have a bright future as wound dressings.^146^

Using an electrospraying approach and subsequent *in situ* cross-linking polymerization, Xu and his group created semi-IPN microspheres by fusing poly (ether sulfone) hydrogels with poly(acrylic acid) (PAA) hydrogels. The microspheres were then given an antibacterial characteristic by immobilizing Ag nanoparticles on the PAA hydrogels. The results revealed highly effective bacterial killing (100% for either *Escherichia coli* or *Staphylococcus aureus*) and removal of positively charged toxins (MB, Cu^2+^, Cd^2+^, and Pb^2+^).^147^

Tables S1, S2, and S3† summarize most of the major results published in the area of IPNs based systems for desalination, dye, and miscellaneous pollutant removal from water.

## 7. Emerging sustainable approaches for water remediation

**Fig. 12:**
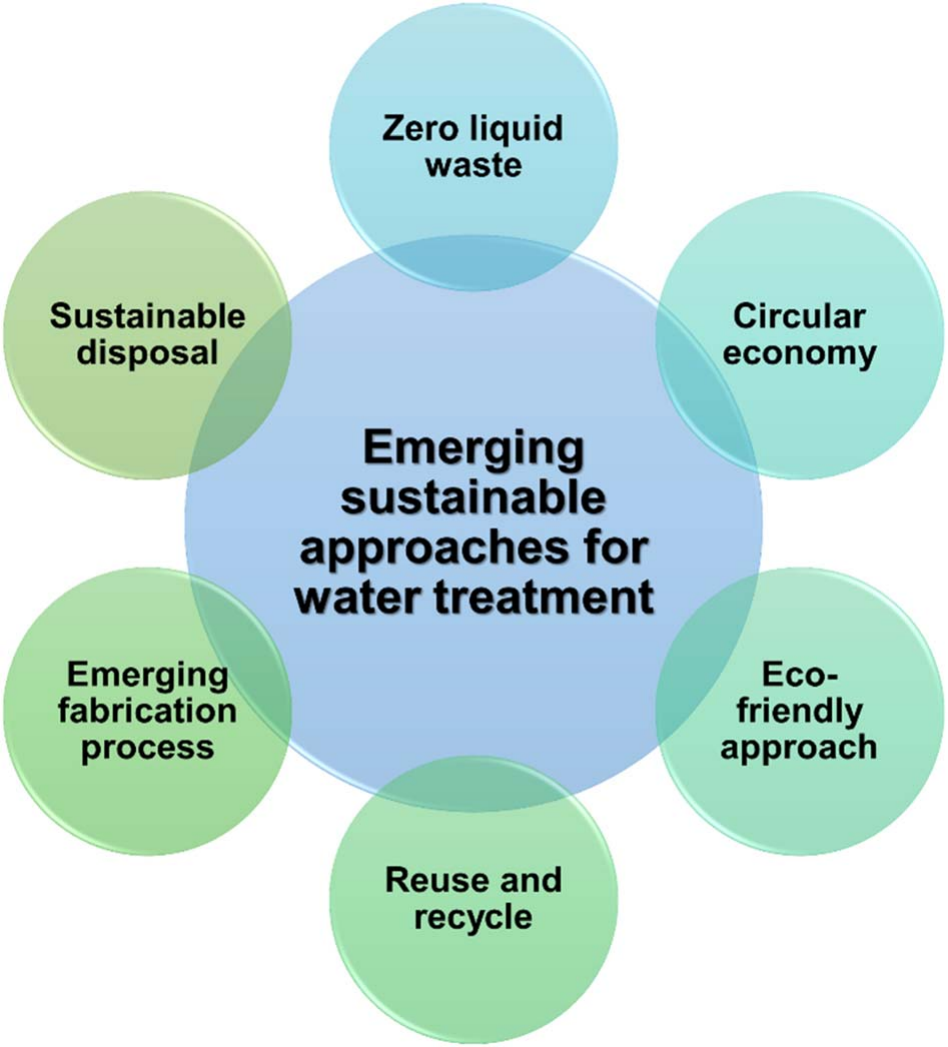
Emerging sustainable approaches for desalination and wastewater treatment.

Desalination and wastewater reuse are two potential options for ensuring water security in many areas across the world. To be considered “sustainable”, these treatment facilities must be cost-efficient, use a renewable resource, be effective on a large scale, be environmentally safe, and be socially acceptable.^33,36,148,149^ However, incorporating IPN in the design of water-remediating systems can provide various benefits for removing pollutants from water. It may impart the chemical, mechanical, and thermal stability and robustness of polymeric material, increasing the system's lifespan and capacity to be reused and recycled without losing its removal efficiency. Additionally, the IPN system is significantly feasible and adaptable in terms of selecting non-toxic chemicals and solvents due to the flexibility of choosing the monomer effectively at the time of IPN design to remove the targeted pollutant. Further, by altering the interpenetrating polymer structure of IPN, it is possible to tailor the membrane pore size and improve antifouling and self-cleaning capabilities. Due to the aforementioned benefits of IPN-based designs features, they can offer cutting-edge sustainable approaches (Fig. 12) like circular economy strategies that emphasize recycling and reuse facilities, zero-liquid discharge (ZLD) due to efficient removal, advanced membrane synthesis approaches, flexibility to select applying non-toxic chemicals and solvents, self-clearing membrane production to increase the longevity of the membrane, *etc.* However, a more intensive investigation is needed to ascertain the sustainability benefits and challenges of IPN-based materials as a future study for emerging innovations.^33,150^

## 8. Summary and prospects

The worldwide freshwater resource shortage has compelled scientists to re-evaluate the supply of clean and safe water. In this situation, desalination and the reuse of wastewater are the major assets to resolve this issue. In this critical review article, we have focused on the application of cutting-edge materials like IPN in environmental restoration, with an emphasis on water treatment and desalination, along with its significance and related issues.

The use of interpenetrating polymer network approaches in the modification of polymeric materials such as membranes, adsorbents including hydrogels is a new approach. After reviewing the current literature, it is observed that IPN-based membranes have several advantages, such as tunable pore size, chemical and mechanical resilience, and reusability. These effectively remove water contaminants including salts, pharmaceuticals, and dyes from polluted water. This structure also has antifouling capabilities because of the compact spacing between polymer chains caused by the densely grafted and interpenetrating polymer networks, which are much smaller than the foulant protein.

The IPN-based membranes in water remediation have a promising future in light of the aforementioned study, but there are still several challenges that need to be resolved at the bench level before the method can be put into practice. To advance the desalination and water treatment, the following factors should be prioritized: field-scale water treatment requires the membrane to resist the water pressure load, and high energy consumption remains to be the fundamental barrier to membrane separation, making industrial-scale membrane production extremely difficult, however, the removal of spent adsorbent from treated water is another difficulty for the adsorbent. This perspective article implies that there is sufficient room for future research on IPNs-based membranes and adsorbents for water remediation to bring it to the industrial level.

## Abbreviations

IPNs: Interpenetrating polymer networks
PVDF: Polyvinylidene fluoride
PVA: Polyvinyl alcohol
PES: Polyether sulfone
PVC: Polyvinyl chloride
PP: Polypropylene
PAN: Polyacrylonitrile
PI: Polyimide
PE: Polyethylene
CA: Cellulose acetate
AA: Acrylic acid
HEMA: Hydroxyethyl methacrylate
PA: Polyamide
XG: Xanthan gum
GO: Graphene oxide
MB: Methylene blue
RB: Rhodamine B
MV: Methyl violet
CV: Crystal violet
MG: Malachite green
APS: Ammonium persulfate

## Conflicts of interest

There are no conflicts of interest to declare.

## Supplementary Material

RA-013-D2RA07843K-s001
