# Supplementary material for: Interpenetrating polymer networks for desalination and water remediation: a comprehensive review of research trends and prospects
Source: RSC Adv. 2023 Feb 20;13(9):6087–107. doi: 10.1039/d2ra07843k (PMC9939980; doi:10.1039/d2ra07843k)
Supplement: RA-013-D2RA07843K-s001 [file RA-013-D2RA07843K-s001.pdf]

Electronic Supplementary Material (ESI) for RSC Advances.
This journal is © The Royal Society of Chemistry 2023

Supporting Information

Interpenetrating polymer networks for desalination and water remediation: A comprehensive review of research trends and prospects

Soumi Dutta, Ria Sen Gupta, Shabnam Pathan, and Suryasarathi Bose*

Department of Materials Engineering, Indian Institute of Science, Bengaluru – 560012, India
*Corresponding Author

Table S1: Desalination using IPN architecture

Sl. No	IPN system	Types of IPN	Desalination application and remarks	References
1	Interpenetrating polymer network of Poly(acrylic acid-co-ethyleneglycol dimethacrylate) (XPAA) and ethoxylated polyethyleneimine (EPEI)	Full IPN	NaCl from water by a thermally reversible (Sirotherm) process using 3000 mg L^{-1}	(Chanda et al., 2010)
2	Hydrogels of N-isopropylacrylamide (NIPAm) in the presence of polysodium acrylate (PSA) or polyvinyl alcohol (PVA)	Semi IPNs	Temperature cycle driven FO desalination at 2000 ppm NaCl concentration	(Cai et al., 2013)
3	Hydrophobic/hydrophilic interpenetrating network composite nanofibers (HH-IPN-CNF) using polyethylene terephthalate (PET) as hydrophobic component and polyvinyl alcohol (PVA)	Full IPN	Water flux of 47.2 LMH and low salt leakage (9.5 gMH) at 0.5 M NaCl solution	(Tian et al., 2014)
4	Cation exchange membrane of polystyrene/PVDF	Semi IPN	96% electrodialysis desalination 2000 ppm	(Lei et al., 2014)
5	Microporous PVDF-PDMS membrane	Semi IPN	99.9% NaCl removal at 30 g L^{-1} concentration	(Sun et al., 2017)

6	PDMS/PVDF membranes	Semi IPNs	99.9% NaCl rejection in vacuum membrane distillation	(Qu et al., 2018)
7	Cation exchange membranes using PVC-St/DVB /SGO based IPN CEM	Full IPN	82% NaCl rejection by electrodialysis.	(Rajput et al., 2018)
8	Polyamide (PA) and poly (N-vinyl-2-pyrrolidone) (PVP)	Semi IPNs	Water flux and salt rejection increased 43.2% and 0.2% (2000 ppm NaCl)	(C. Wang et al., 2021)
9	MOF-incorporated Cu-based alginate/PVA hydrogel (MOF-Alg(Cu)/PVA) beads	Full IPN	43% NaCl at 10000 ppm by Ion adsorption desalination technique	(Lee et al., 2021)
10	Crosslinked poly(2-acrylamido-2-methyl-l-propanesulfonic acid-co-acrylamide) (P(AMPS-AM)) and linear polyelectrolyte polyacrylic acid (PAA)	Semi IPNs	Water flux of 2.20 L m^{-2} h^{-1} (LMH) from 2000 ppm NaCl solution	(Xu et al., 2022)

Table S2: Dye removal using IPN architecture

Sl. No.	IPN system	Type of IPN	Dye removal efficiency and remarks	References
1.	IPN gel beads from SA, MAPTAC (3-(methacrylamido) propyl trimethyl ammonium chloride) and/or AM (acrylamide)	Semi IPN	Adsorption of cationic dye Taiacryl Brilliant Red 4GN: 21 mg g-1	(Kusuktham, 2006)
2.	Sodium alginate/acrylamidesemi-interpenetrating polymer networks ((NaAlg/AAm)IPN)	Semi IPN	Adsorption capacity followed the order: Magenta< Safranine-O<Methylene Blue< Methyl Violet	(Şolpan et al., 2008)
3.	Polystyrene/polyamide macroporous crosslinked IPN	Full IPN	Adsorption of quercetin pigment: 8.6 mg mL^{-1}	(Liao et al., 2010)
4.	poly (acrylamide-co-acrylic acid) and polyvinylalcohol hydrogels	Semi IPN	MB: Removal 95%	(Zendehdel et al., 2010)
5.	Cross-Linked Chitosan-Poly(acrylamide) Adsorbent Hydrogels	Full IPN	Adsorption of EY-4GL: 64.5 mg g^{-1} and S-Blue: 38.7 mg g^{-1}	(Ekici et al., 2011)
6.	Hydrogel from polyvinyl alcohol and poly (acrylic acid-co-hydroxyethylmethacrylate) copolymer	Semi IPN and Full IPN	Maximum adsorption capacity for Rhodamine B: 2.9 mg g^{-1}, Methyl violet: 3.7 mg g^{-1}	(Mandal et al., 2012)
7.	Hydrogels of PEG and (AAm/SMA) copolymer of acrylamide/sodium methacrylate	Semi IPN	Janus Green B dye: Removal 90%	(Üzüm & Karadağ, 2012)
8.	Polymethacrylic acid grafted cellulose–bentonite (PMAA-g-	Full IPN	MB: removal 99.9%	(Anirudhan & Tharun, 2012)

	Cell/Bent) IPN			
9.	Hhydrogels from PEG (polyethylene glycol) and Aam (acrylamide)	Semi IPN	Adsorption capacities of acid red 17: 342.54 mg g^{-1}, acid orange 7: 221.1 mg g^{-1} and methyl orange: 185.24 mg g^{-1}	(S. Zhao et al., 2012)
10.	Hydrogels based on polyacrylamide and chitosan	Semi and Full IPN	High anionic dye adsorption capacity of direct blue 1: 2.804 mg g^{-1} by semi IPN and high cationic dye adsorption of MB: 6.744 mg g^{-1} by full IPN	(Dragan et al., 2012)
11.	Carboxymethylcellulose/ poly(acrylamide-co-hydroxyethyl methacrylate) hydrogel	Semi IPN	Maximum adsorption capacity of Methyl violet (MV): 450 mg g^{-1}, Basic Fuchsin: 350 mg g^{-1}	(Bhattacharyya & Ray, 2013)
12.	Poly(acrylamide-aniline)-grafted gum ghatti based crosslinked conducting hydrogel	Semi IPN	MG dye removal 98% after 20 hrs of operation	(K. Sharma et al., 2013)
13.	Full IPN of polymethacrylic acid and chitosan	Full IPN	MV: 91% and CR: 82% removal	(Maity & Ray, 2014)
14.	Poly(ether amine) (hPEA)/Poly(vinyl alcohol) (PVA) hyperbranched Interpenetrating Network (IPN)	Full IPN	Fluorescein dyes: Removal 90%	(P. Zhang et al., 2014)
15.	Semi-interpenetrating polymer network sodium alginate and isopropyl acrylamide	Semi IPN	600 mg g-1 adsorption of RB 4 (Reactive Blue) dye	(Dhanapal & Subramanian, 2014)

16.	IPN hydrogel from acrylic copolymer and chitosan	Full IPN	98% CR and 94% MV dye removal	(Mandal & Ray, 2014)
17.	P(NIPAM-MBAM) hydrogel microspheres (poly(N-isopropylacrylamide) (PNIPAM) and poly(methacrylic acid)	Full IPN	3.7 mg g-1 Magenta and 0.7 mg g-1 CR adsorption	(Ahmad et al., 2014)
18.	Semi IPN hydrogels from GEL (gelatin) and/or PEG (poly (ethylene glycol)) with AAm (acrylamide) and 4-styrenesulfonic acid sodium salt	Semi IPN	93.34% MV dye adsorption percentage	(Kundakci & Karadağ, 2014)
19.	TiO2 nanoparticle immobilized Sodium alginate-polymethacrylic acid hydrogel	Semi IPN	MB: Removal 93%	(Lučić Škorić et al., 2015)
20.	Acrylic acid-2-hydroxyethyl methacrylate IPN hydrogels	Semi and Full IPN	>99% MV and >93% removal of MV and Fuchsine dye	(Bera et al., 2015)
21.	In-situ crosslinked PES (polyethersulfone) and modified chitosan hydrogel adsorbent	Semi IPN	CR dye: Removal 80%	(R. Wang et al., 2015)
22.	Guar gum/acrylic acid hydrogel (Ggum-cl-poly(AA-ipn-aniline)) IPN	Semi IPN	MB: Nearly 94% and demonstrated antibacterial activity	(R. Sharma, Kaith, et al., 2015)
23.	Chitosan/gelatin porous materials	Full IPN	Acid orange II maximum adsorption capacity 573 mg g^{-1}	(Cui et al., 2015)

24.	Ggum-cl-poly(IA) crosslinked hydrogel (itaconic acid and Guaran polysaccharide)	Semi IPN	Removal 84.5% and 81% of MB dye in neutral and acidic pH, respectively	(R. Sharma, Kalia, et al., 2015)
25.	IPN hydrogels based on chitosan (CS) and poly(acrylic acid) (PAA)	Semi IPN	Adsorption capacity of basic BB12 (Nil Blue) dye: 430 mg g^{-1}	(J. Wang & Li, 2015)
26.	Semi IPN hydrogel from chitosan and starch	Semi IPN	Adsorption capacity of DR 80 dye: 312.77 mg g^{-1}	(Ngwabebhoh et al., 2016)
27.	Porous chitosan/hydroxyapatite composite membrane	Sequential IPN	DB (direct black): Removal 98%	(Shi et al., 2017)
28.	Guar gum-g-(acrylic acid-co-acrylamide-co-3-acrylamido propanoic acid) hydrogel	Full IPN	Maximum adsorption of MB: 27.06 mg g^{-1} and SF (safranine F) dye: 39.35 mg g^{-1}, and effective heavy metal removal	(Singha et al., 2017)
29.	Xanthan gum, PVA and tartaric acid-based semi IPN hydrogel	Semi IPN	RB: 70% and AO (auramine-O): 63% removal	(Sukriti et al., 2017)
30.	Aloe vera–acrylic acid-co-acrylamide	Semi IPN	Malachite green (MG): Removal 94%	(Saruchi et al., 2018)
31.	Av-cl-poly(AA-ipn-AAm) (aloe vera, acrylic acid, acrylamide IPN) microwave assisted	Semi IPN	MG: Removal 97.3%	(V. Kumar et al., 2018)
32.	Poly(acrylic acid)/poly(vinyl alcohol)/yeast superabsorbent polymers (PAA/PVA/yeast SAPs)	Semi IPN	Maximum adsorption capacity of 50 mg g^{-1} for MB	(Feng et al., 2018)

33.	Hydrogel from (gum copal alcohols-collagen)-co-poly(acrylamide) and acrylic acid	Full IPN	Adsorption capacity of MB: 1.7 mg g^{-1}	(Kaur & Jindal, 2018)
34.	Hydrogel based on gum copal-collagen (GcA-coll)	Full IPN	MG (Malachite Green) dye: removal 88%	(Kaur et al., 2018)
35.	QPVA (quartarnized PVA)/acrylamide full IPN alkaline membranes	Full IPN	CR removal better than RB in binary dye systems and single system	(J. Wang et al., 2018)
36.	PVA/CNC (cellulose nanocrystal)/polyHEMA hydrogels	Full IPN	MB: 91% and (xylenol orange) XO: 93% removal	(Bai et al., 2018)
37.	Cellulose filament/poly(NIPAM-co-AAc) hybrid hydrogels	Semi IPN	MV dye: Removal 226.02 mg g^{-1}	(M. Zhang et al., 2018)
38.	Gum acacia/sodium alginate hydrogel	Semi IPN	MG dye: removal 95.39%, CV (crystal violet): 94.56% and AO (auramine-O): 97.49%	(A. K. Sharma et al., 2019)
39.	N,N,N-trimethyl chitosan (TMC) and xanthan gum (XG) hydrogel	Full IPN	CV dye: removal 94.4 % with antibacterial activity	(Abu Elella et al., 2019)
40.	Sodium humate/poly(acrylamide-co-methacrylic acid)/kaolin semi-interpenetrating polymer network hybrid hydrogel	Hybrid IPN	Adsorption of MB dye: 833.33 mg g^{-1}	(Yilmaz et al., 2019)
41.	Carboxymethyl cellulose/poly(acrylic acid) interpenetrating polymer network hydrogels	Full IPN	Adsorption of MB dye: 613 mg g^{-1}	(Toledo et al., 2019)

42.	PAA-XG-GO Semi IPN nanocomposite (cross-linked poly acrylic acid/xanthan gum/graphene oxide)	Semi IPN	MB: Removal 88.5% and swelling upto 485%	(Hosseini et al., 2020)
43.	Graphene oxide decorated superporous polyacrylamide hydrogel (MCC/poly (AAm-co-NaAc)/r-GO (L))	Full IPN	MB: 98% and RB: 97.6% removed	(Sarkar et al., 2020)
44.	PVA/KHA/GG Hydrogel (polyvinyl alcohol/potassium humate/guar gum)	Semi IPN	Maximum adsorption capacity for MB dye: 1166.73 mg g^{-1} and Pb(II): 625.21 mg g^{-1}	(Niu et al., 2020)
45.	Cassava starch-graft-poly(acrylamide) hydrogel	Full IPN	>85% of MB removal within less than 10 hrs	(Junlapong et al., 2020)
46.	Copolymer poly(di(ethylene glycol) methyl ether methacrylate-co-poly(ethylene glycol) methyl ether methacrylate) (P(MEO2MA-co-OEGMA300)) in alginate–Ca2+ hydrogel	Hybrid IPN	MB: Removal 96%	(N. Hu et al., 2020)
47.	Poly(vinyl alcohol-g-acrylamide)/SiO_2@ZnO photocatalytic hydrogel composite	Semi IPN	Maximum adsorption capacity for MB: 757 mg g^{-1}	(Maijan et al., 2020)
48.	Starch-grafted poly(N,N-dimethyl acrylamide) hydrogel	Full IPN	Acid Red 8: removal 91%	(Sadik et al., 2020)
49.	Self-supported gel filter membrane with Ca^{2+} alginate network and covalently crosslinked	Full IPN	MB: removal 93% and DR: removal 95%	(J. Hu et al., 2020)

	polyacrylamide network			
50.	Polyvinyl Alcohol−Alginate/Bentonite Semi-Interpenetrating Polymer Network Nanocomposite Hydrogel Beads	Semi IPN	Maximum adsorption capacity of MB: 51.34 mg g^{-1}	(Aljar et al., 2021)
51.	KG-Gl-PVA Semi IPN Microspheres (katira gum/polyvinyl alcohol)	Semi IPN	96.92% of Bismark brown-yellow dye removal	(A. Kumar et al., 2021)
52.	Solid state liquid crystal shell IPN	IPN via microfluidic method	MB and AR37: Removal 99%	(Gwon & Park, 2021)
53.	Hydroxyl-terminated polybutadiene (HTPB) and multifunctional isocyanate (MFI) membrane	Semi IPN	MB and MO dye removal 98%	(Nozad et al., 2022)
54.	PVDF/PDA IPN membrane	Sequential IPN	>97% removal of MB and CR dyes	(Sen Gupta et al., 2022)
55.	Pullulan/PDA Semi IPN hydrogels (sPDA)	Semi IPN	Maximum adsorption capacity of 107 mg g^{-1} for Crystal violet (CV)	(Wu et al., 2022)
56.	Poly(vinyl alcohol)/partially hydrolyzed polyacrylamide/graphene oxide Semi IPN nanocomposite hydrogel	Semi IPN	Maximum adsorption capacity of 714.8 mg g^{-1} at 30°C for MB dye	(Rahmatpour et al., 2022)
57.	TFC semi IPN membrane from 3, 5-diaminobenzoic acid (DABA) and piperazine (PIP)	Semi IPN	MB: removal 99%	(Waheed et al., 2022)

58.	PVDF-Pd (palladium)/ Semi IPN composite membrane	Semi IPN	CR and DB dyes removal >99%	(Zhai et al., 2022)
59.	Semi-interpenetrating network based on xanthan gum-cl-2-(N-morpholinoethyl methacrylate)/titanium oxide	Semi IPN	Adsorption capacity of MB: 63.34 mg g^{-1} and CV: 83.25 mg g^{-1}	(Taktak & Özyaranlar, 2022)
60.	Semi IPN hydrogels based on acrylamide (AAm) and itaconic acid (ITA)	Semi IPN	Adsorption capacity of MB: 15 mg g^{-1} and MG: 8 mg g^{-1}	(Ciftbudak & Orakdogen, 2022)
61.	Poly(N-isopropylacrylamide-co-methacrylic acid) P(NIPA-MA) gels	Semi IPN	MV(methyl violet): removal 75%	(Kalkan & Orakdogen, 2022)
62.	Chitosan- and Alginate-Based Hydrogels	Semi IPN	MB dye removal 96% within 10 mins	(ALSamman & Sánchez, 2022)
63.	Cellulose acetate/acrylic acid-glutaraldehyde semi-interpenetrating networks	Semi IPN	MB: removal 90%	(Rana et al., 2022)
64.	Methylcellulose/tannic acid complex particles coated on alginate hydrogel scaffold	IPN via Pickering emulsion	MB dye adsorption: 791.17 mg g^{-1}	(Abebe & Kim, 2022)

Table S3: Miscellaneous water remediation application using IPNs based material				
**Sl. No**	**IPN system**	**Types of IPN**	**Miscellaneous application in water remediation and remarks**	**References**
1	Sodium alginate/acrylamide) based IPN	Semi IPN	Detection of Ni^{2+}, Cd^{2+}, and Pb^{2}	(Şolpan & Torun, 2005)
2	Starch/acryl amide-based hydrogels	Full IPN	adsorption of heavy metal ions like Cu^{2+} and Ni^{2+} adsorption	(Peñaranda A. & Sabino, 2010)
3	Poly(polyethylene glycol diacrylate) poly(PEGDA) and poly(methacrylic acid) (PMAA) IPN hydrogel	Full IPN	Cu(II), Cd(II), or Pb(II) ion solutions	(J. Wang et al., 2011)
4	Polyvinyl alcohol/poly (acrylic acid-co-acrylic amide) (PVA-P(AA-co-AM)) hydrogels	Semi IPN	Cobalt (II) adsorption	(X. Wang et al., 2016)
5	IPN of acrylamide (AAm) and 1,4-butanediol vinyl ether (BVE)	Full IPN	Adsorption and determination of Cu^{2+}, Ni^{2+} and Zn^{2+} ions	(J. Wang et al., 2016)
6	2-hydroxyethyl methacrylate (HEMA), acrylamide (AM), polyvinyl alcohol (PVA) and chitosan (CS)	Full IPN	Excellent antibacterial activity against E. *coli*	(Panpinit et al., 2020)
7	interpenetrating polymeric networks (IPN) based on sodium alginate, carrageenan and bentonite	Full IPN	Excellent adsorption efficiency of methylene blue, Fe^{3+}, Ni^{2+}, and Cr^{3+} ions is 1271, 1550, 1500 and 1540 mg/g adsorbent, respectively.	(Al-Sakkari et al., 2020)
8	Chitosan/polyacrylamide IPN modified with α-ketoglutaric acid	Semi IPN	More than 90% selective adsorption for Cu(II), Pb(II), and Zn(II) in a mixture of heavy metal ions upto five adsorption-desorption cycles	(Z. Zhao et al., 2021)

9	Poly(2-hydroxyethyl methacrylate-co-acrylamide)/poly(vinyl alcohol) (P(HEMA-co-AM)/PVA) IPN hydrogels	Full IPN	Cu(II) and Pb(II) ions adsorption	(Tanan et al., 2021)
10	Chitosan/gelatin-based IPN incorporated with melanin-coated titania hollow nanospheres (CG@MPT-h)	Full IPN	Solar-Driven Wastewater Treatment	(X. Wang et al., 2021)
11	Cellulose Nanofibril/Chitosan IPN hydrogel crosslinked by Fluorescent carbon dots hydrogel	Full IPN	Simultaneously detection and adsorbent of Cu(II) and Cr(VI) in water.	(Chen et al., 2022)
12	Thermo-responsive Semi IPN/PVDF@Pd bilayer composite membrane	Semi IPN	Water flux 27.0 L m^{-2} h^{-1} bar^{-1} and 99.74% adsorption of p-nitrophenol	(S. Wang et al., 2022)

References

Abebe, M. W., & Kim, H. (2022). Methylcellulose/tannic acid complex particles coated on alginate hydrogel scaffold via Pickering for removal of methylene blue from aqueous and quinoline

from non-aqueous media. *Chemosphere*, *286*, 131597. https://doi.org/10.1016/j.chemosphere.2021.131597

Abu Elella, M. H., ElHafeez, E. A., Goda, E. S., Lee, S., & Yoon, K. R. (2019). Smart bactericidal filter containing biodegradable polymers for crystal violet dye adsorption. *Cellulose*, *26*(17), 9179–9206. https://doi.org/10.1007/s10570-019-02698-1

Ahmad, H., Nurunnabi, M., Rahman, M. M., Kumar, K., Tauer, K., Minami, H., & Gafur, M. A. (2014). Magnetically doped multi stimuli-responsive hydrogel microspheres with IPN structure and application in dye removal. *Colloids and Surfaces A: Physicochemical and Engineering Aspects*, *459*, 39–47. https://doi.org/10.1016/j.colsurfa.2014.06.038

Aljar, M. A. A., Rashdan, S., & Abd El-Fattah, A. (2021). Environmentally Friendly Polyvinyl Alcohol−Alginate/Bentonite Semi-Interpenetrating Polymer Network Nanocomposite Hydrogel Beads as an Efficient Adsorbent for the Removal of Methylene Blue from Aqueous Solution. *Polymers*, *13*(22), 4000. https://doi.org/10.3390/polym13224000

Al-Sakkari, E. G., Abdeldayem, O. M., Genina, E. E., Amin, L., Bahgat, N. T., Rene, E. R., & El-Sherbiny, I. M. (2020). New alginate-based interpenetrating polymer networks for water treatment: A response surface methodology based optimization study. *International Journal of Biological Macromolecules*, *155*, 772–785. https://doi.org/10.1016/j.ijbiomac.2020.03.220

ALSamman, M. T., & Sánchez, J. (2022). Chitosan- and Alginate-Based Hydrogels for the Adsorption of Anionic and Cationic Dyes from Water. *Polymers*, *14*(8), 1498. https://doi.org/10.3390/polym14081498

Anirudhan, T. S., & Tharun, A. R. (2012). Preparation and adsorption properties of a novel interpenetrating polymer network (IPN) containing carboxyl groups for basic dye from aqueous media. *Chemical Engineering Journal*, *181–182*, 761–769. https://doi.org/10.1016/j.cej.2011.11.077

Bai, H., Li, Z., Zhang, S., Wang, W., & Dong, W. (2018). Interpenetrating polymer networks in polyvinyl alcohol/cellulose nanocrystals hydrogels to develop absorbent materials. *Carbohydrate Polymers*, *200*, 468–476. https://doi.org/10.1016/j.carbpol.2018.08.041

Bera, R., Dey, A., Datta sarma, A., & Chakrabarty, D. (2015). Synthesis and characterization of acrylic acid-2-hydroxyethyl methacrylate IPN hydrogels. *RSC Advances*, *5*(93), 75870–75880. https://doi.org/10.1039/C5RA12110H

Bhattacharyya, R., & Ray, S. K. (2013). Kinetic and equilibrium modeling for adsorption of textile dyes in aqueous solutions by carboxymethyl cellulose/poly(acrylamide- *co* -hydroxyethyl methacrylate) semi-interpenetrating network hydrogel. *Polymer Engineering & Science*, *53*(11), 2439–2453. https://doi.org/10.1002/pen.23501

Cai, Y., Shen, W., Loo, S. L., Krantz, W. B., Wang, R., Fane, A. G., & Hu, X. (2013). Towards temperature driven forward osmosis desalination using Semi IPN hydrogels as reversible draw agents. *Water Research*, *47*(11), 3773–3781. https://doi.org/10.1016/j.watres.2013.04.034

Chanda, M., Pillay, S. A., Sarkar, A., & Modak, J. M. (2010). Interpenetrating Networks of Cross-Linked Poly(acrylic acid) and Cross-linked Polyethyleneimine (80% Ethoxylated) for Desalination of Brackish Water by Thermoreversible Sorption. *Industrial & Engineering Chemistry Research*, *49*(16), 7136–7146. https://doi.org/10.1021/ie100342r

Chen, X., Song, Z., Yuan, B., Li, X., Li, S., Thang Nguyen, T., Guo, M., & Guo, Z. (2022). Fluorescent carbon dots crosslinked cellulose Nanofibril/Chitosan interpenetrating hydrogel system for sensitive detection and efficient adsorption of Cu (II) and Cr (VI). *Chemical Engineering Journal*, *430*, 133154. https://doi.org/10.1016/j.cej.2021.133154

Ciftbudak, S., & Orakdogen, N. (2022). Assessing the compressive elasticity and multi-responsive property of gelatin-containing weakly anionic copolymer gels *via* Semi IPN strategy. *Soft Matter*, *18*(37), 7181–7200. https://doi.org/10.1039/D2SM00938B

Cui, L., Xiong, Z., Guo, Y., Liu, Y., Zhao, J., Zhang, C., & Zhu, P. (2015). Fabrication of interpenetrating polymer network chitosan/gelatin porous materials and study on dye adsorption properties. *Carbohydrate Polymers*, *132*, 330–337. https://doi.org/10.1016/j.carbpol.2015.06.017

Dhanapal, V., & Subramanian, K. (2014). Recycling of reactive dye using semi-interpenetrating polymer network from sodium alginate and isopropyl acrylamide. *Journal of Applied Polymer Science*, *131*(21). https://doi.org/10.1002/app.40968

Dragan, E. S., Perju, M. M., & Dinu, M. V. (2012). Preparation and characterization of IPN composite hydrogels based on polyacrylamide and chitosan and their interaction with ionic dyes. *Carbohydrate Polymers*, *88*(1), 270–281. https://doi.org/10.1016/j.carbpol.2011.12.002

Ekici, S., Güntekin, G., & Saraydin, D. (2011). The Removal of Textile Dyes with Cross-Linked Chitosan-Poly(acrylamide) Adsorbent Hydrogels. *Polymer - Plastics Technology and Engineering*, *50*(12), 1247–1255. https://doi.org/10.1080/03602559.2011.574674

Feng, D., Bai, B., Wang, H., & Suo, Y. (2018). Novel Fabrication of PAA/PVA/Yeast Superabsorbent with Interpenetrating Polymer Network for pH-Dependent Selective Adsorption of Dyes. *Journal of Polymers and the Environment*, *26*(2), 567–588. https://doi.org/10.1007/s10924-017-0972-y

Gwon, S., & Park, S. (2021). Preparation of uniformly sized interpenetrating polymer network polyelectrolyte hydrogel droplets from a solid-state liquid crystal shell. *Journal of Industrial and Engineering Chemistry*, *99*, 235–245. https://doi.org/10.1016/j.jiec.2021.04.032

Hosseini, S. M., Shahrousvand, M., Shojaei, S., Khonakdar, H. A., Asefnejad, A., & Goodarzi, V. (2020). Preparation of superabsorbent eco-friendly semi-interpenetrating network based on cross-linked poly acrylic acid/xanthan gum/graphene oxide (PAA/XG/GO): Characterization and dye removal ability. *International Journal of Biological Macromolecules*, *152*, 884–893. https://doi.org/10.1016/j.ijbiomac.2020.02.082

Hu, J., Chen, Y., Lu, J., Fan, X., Li, J., Li, Z., Zeng, G., & Liu, W. (2020). A self-supported gel filter membrane for dye removal with high anti-fouling and water flux performance. *Polymer*, *201*, 122531. https://doi.org/10.1016/j.polymer.2020.122531

Hu, N., Chen, C., Tan, J., Wang, W., Wang, C., Fan, H., Wang, J., Müller-Buschbaum, P., & Zhong, Q. (2020). Enhanced Adsorption of Methylene Blue Triggered by the Phase Transition of Thermoresponsive Polymers in Hybrid Interpenetrating Polymer Network Hydrogels. *ACS Applied Polymer Materials*, *2*(8), 3674–3684. https://doi.org/10.1021/acsapm.0c00661

Junlapong, K., Maijan, P., Chaibundit, C., & Chantarak, S. (2020). Effective adsorption of methylene blue by biodegradable superabsorbent cassava starch-based hydrogel. *International Journal of Biological Macromolecules*, *158*, 258–264. https://doi.org/10.1016/j.ijbiomac.2020.04.247

Kalkan, B., & Orakdogen, N. (2022). Strength and salt/pH dependent-sorption capacity modulation of N-(alkyl)acrylamide-based Semi IPN hybrid gels reinforced with silica nanoparticles. *European Polymer Journal*, *173*, 111296. https://doi.org/10.1016/j.eurpolymj.2022.111296

Kaur, S., & Jindal, R. (2018). Synthesis of interpenetrating network hydrogel from (gum copal alcohols-collagen)-co-poly(acrylamide) and acrylic acid: Isotherms and kinetics study for removal of methylene blue dye from aqueous solution. *Materials Chemistry and Physics*, *220*, 75–86. https://doi.org/10.1016/j.matchemphys.2018.08.008

Kaur, S., Jindal, R., & Kaur Bhatia, J. (2018). Synthesis and RSM-CCD optimization of microwave-induced green interpenetrating network hydrogel adsorbent based on gum copal for selective removal of malachite green from waste water. *Polymer Engineering and Science*, *58*(12), 2293–2303. https://doi.org/10.1002/pen.24851

Kumar, A., Goel, A., & others. (2021). KG-Gl-PVA Semi IPN Microspheres: Synthesis, Characterisation, BBY Adsorption And Potential Applications. *NVEO-NATURAL VOLATILES & ESSENTIAL OILS Journal| NVEO*, 10124–10153.

Kumar, V., Rehani, V., Kaith, B. S., & Saruchi, S. (2018). Synthesis of a biodegradable interpenetrating polymer network of Av-cl-poly(AA-ipn-AAm) for malachite green dye removal: Kinetics and thermodynamic studies. *RSC Advances*, *8*(73), 41920–41937. https://doi.org/10.1039/C8RA07759B

Kundakci, S., & Karadağ, E. (2014). Preliminary swelling and dye sorption studies of acrylamide/4-styrenesulfonic acid sodium salt copolymers and semi-interpenetrating polymer networks composed of gelatin and/or PEG. *Polymer Bulletin*, *71*(2), 351–370. https://doi.org/10.1007/s00289-013-1065-2

Kusuktham, B. (2006). Preparation of interpenetrating polymer network gel beads for dye absorption. *Journal of Applied Polymer Science*, *102*(2), 1585–1591. https://doi.org/10.1002/app.23882

Lee, S. J., Lim, H. W., & Park, S. H. (2021). Adsorptive seawater desalination using MOF-incorporated Cu-alginate/PVA beads: Ion removal efficiency and durability. *Chemosphere*, *268*, 128797. https://doi.org/10.1016/j.chemosphere.2020.128797

Lei, Y., Luo, Y., Chen, F., & Mei, L. (2014). Sulfonation Process and Desalination Effect of Polystyrene/PVDF Semi-Interpenetrating Polymer Network Cation Exchange Membrane. *Polymers*, *6*(7), 1914–1928. https://doi.org/10.3390/polym6071914

Liao, W., Gao, S., Xie, X., & Xu, M. (2010). Macroporous crosslinked hydrophobic/hydrophilic polystyrene/polyamide interpenetrating polymer network: Synthesis, characterization, and adsorption behaviors for quercetin from aqueous solution. *Journal of Applied Polymer Science*, *118*(6), 3643–3648. https://doi.org/10.1002/app.32479

Lučić Škorić, M., Milosavljević, N., Radetić, M., Šaponjić, Z., Radoičić, M., & Kalagasidis Krušić, M. (2015). Synthesis and characterization of interpenetrating polymer network based on sodium alginate and methacrylic acid and potential application for immobilization of TiO_2 nanoparticles. *Polymer Engineering & Science*, *55*(11), 2511–2518. https://doi.org/10.1002/pen.24141

Maijan, P., Amornpitoksuk, P., & Chantarak, S. (2020). Synthesis and characterization of poly(vinyl alcohol-g-acrylamide)/SiO2@ZnO photocatalytic hydrogel composite for removal and degradation of methylene blue. *Polymer*, *203*, 122771. https://doi.org/10.1016/j.polymer.2020.122771

Maity, J., & Ray, S. K. (2014). Enhanced adsorption of methyl violet and congo red by using semi and full IPN of polymethacrylic acid and chitosan. *Carbohydrate Polymers*, *104*, 8–16. https://doi.org/10.1016/j.carbpol.2013.12.086

Mandal, B., & Ray, S. K. (2014). Swelling, diffusion, network parameters and adsorption properties of IPN hydrogel of chitosan and acrylic copolymer. *Materials Science and Engineering: C*, *44*, 132–143. https://doi.org/10.1016/j.msec.2014.08.021

Mandal, B., Ray, S. K., & Bhattacharyya, R. (2012). Synthesis of full and semi Interpenetrating hydrogel from polyvinyl alcohol and poly (acrylic acid-co-hydroxyethylmethacrylate) copolymer: Study of swelling behavior, network parameters, and dye uptake properties. *Journal of Applied Polymer Science*, *124*(3), 2250–2268. https://doi.org/10.1002/app.35298

Ngwabebhoh, F. A., Gazi, M., & Oladipo, A. A. (2016). Adsorptive removal of multi-azo dye from aqueous phase using a Semi IPN superabsorbent chitosan-starch hydrogel. *Chemical Engineering Research and Design*, *112*, 274–288. https://doi.org/10.1016/j.cherd.2016.06.023

Niu, Y., Han, X., Huang, L., & Song, J. (2020). Methylene Blue and Lead(II) Removal via Degradable Interpenetrating Network Hydrogels. *Journal of Chemical & Engineering Data*, *65*(4), 1954–1967. https://doi.org/10.1021/acs.jced.9b01134

Nozad, E., Poursattar Marjani, A., & Mahmoudian, M. (2022). A novel and facile Semi IPN system in fabrication of solvent resistant nano-filtration membranes for effective separation of dye contamination in water and organic solvents. *Separation and Purification Technology*, *282*, 120121. https://doi.org/10.1016/j.seppur.2021.120121

Panpinit, S., Pongsomboon, S., Keawin, T., & Saengsuwan, S. (2020). Development of multicomponent interpenetrating polymer network (IPN) hydrogel films based on 2-hydroxyethyl methacrylate (HEMA), acrylamide (AM), polyvinyl alcohol (PVA) and chitosan (CS) with enhanced mechanical strengths, water swelling and antibacterial properties. *Reactive and Functional Polymers*, *156*, 104739. https://doi.org/10.1016/j.reactfunctpolym.2020.104739

Peñaranda A., J. E., & Sabino, M. A. (2010). Effect of the presence of lignin or peat in IPN hydrogels on the sorption of heavy metals. *Polymer Bulletin*, *65*(5), 495–508. https://doi.org/10.1007/s00289-010-0264-3

Qu, Z.-T., Duan, S.-Y., Li, B.-B., Sun, D., & Gu, Y.-L. (2018). PDMS/PVDF microporous membrane with semi-interpenetrating polymer networks for vacuum membrane distillation. *Journal of Applied Polymer Science*, *135*(8), 45792. https://doi.org/10.1002/app.45792

Rahmatpour, A., Soleimani, P., & Mirkani, A. (2022). Eco-friendly poly(vinyl alcohol)/partially hydrolyzed polyacrylamide/graphene oxide Semi IPN nanocomposite hydrogel as a reusable and efficient adsorbent of cationic dye methylene blue from water. *Reactive and Functional Polymers*, *175*, 105290. https://doi.org/10.1016/j.reactfunctpolym.2022.105290

Rajput, A., Yadav, V., Sharma, P. P., & Kulshrestha, V. (2018). Synthesis of SGO composite interpenetrating network (CIPN) cation exchange membranes: Stability and salt removal efficiency. *Journal of Membrane Science*, *564*, 44–52. https://doi.org/10.1016/j.memsci.2018.07.004

Rana, J., Goindi, G., & Kaur, N. (2022). Potential prospects of cellulose acetate/acrylic acid-glutaraldehyde semi-interpenetrating networks to remove methylene blue dye from wastewater. *Materials Today: Proceedings*, S2214785322052944. https://doi.org/10.1016/j.matpr.2022.08.127

Sadik, W. A.-A., El-Demerdash, A.-G. M., Abbas, R., & Gabre, H. A. (2020). Fast synthesis of an eco-friendly starch-grafted poly(N,N-dimethyl acrylamide) hydrogel for the removal of Acid Red 8 dye from aqueous solutions. *Polymer Bulletin*, *77*(8), 4445–4468. https://doi.org/10.1007/s00289-019-02958-x

Sarkar, N., Sahoo, G., & Swain, S. K. (2020). Reduced graphene oxide decorated superporous polyacrylamide based interpenetrating network hydrogel as dye adsorbent. *Materials Chemistry and Physics*, *250*, 123022. https://doi.org/10.1016/j.matchemphys.2020.123022

Saruchi, Kumar, V., Rehani, V., & Kaith, B. S. (2018). Microwave-assisted synthesis of biodegradable interpenetrating polymer network of aloe vera–poly(acrylic acid-co-acrylamide) for removal of malachite green dye: Equilibrium, kinetics and thermodynamic studies. *Iranian Polymer Journal*, *27*(11), 913–926. https://doi.org/10.1007/s13726-018-0665-y

Sen Gupta, R., Padmavathy, N., Agarwal, P., & Bose, S. (2022). PH-triggered bio-inspired membranes engineered using sequential interpenetrating polymeric networks for tunable antibiotic and dye removal. *Chemical Engineering Journal*, *446*. https://doi.org/10.1016/j.cej.2022.136997

Sharma, A. K., Priya, Kaith, B. S., Sharma, N., Bhatia, J. K., Tanwar, V., Panchal, S., & Bajaj, S. (2019). Selective removal of cationic dyes using response surface methodology optimized gum acacia-sodium alginate blended superadsorbent. *International Journal of Biological Macromolecules*, *124*, 331–345. https://doi.org/10.1016/j.ijbiomac.2018.11.213

Sharma, K., Kaith, B. S., Kumar, V., Kumar, V., Som, S., Kalia, S., & Swart, H. C. (2013). Synthesis and properties of poly(acrylamide-aniline)-grafted gum ghatti based nanospikes. *RSC Advances*, *3*(48), 25830. https://doi.org/10.1039/c3ra44809f

Sharma, R., Kaith, B. S., Kalia, S., Pathania, D., Kumar, A., Sharma, N., Street, R. M., & Schauer, C. (2015). Biodegradable and conducting hydrogels based on Guar gum polysaccharide for antibacterial and dye removal applications. *Journal of Environmental Management*, *162*, 37–45. https://doi.org/10.1016/j.jenvman.2015.07.044

Sharma, R., Kalia, S., Kaith, B. S., Pathania, D., Kumar, A., & Thakur, P. (2015). Guaran-based biodegradable and conducting interpenetrating polymer network composite hydrogels for adsorptive removal of methylene blue dye. *Polymer Degradation and Stability*, *122*, 52–65. https://doi.org/10.1016/j.polymdegradstab.2015.10.015

Shi, C., Lv, C., Wu, L., & Hou, X. (2017). Porous chitosan/hydroxyapatite composite membrane for dyes static and dynamic removal from aqueous solution. *Journal of Hazardous Materials*, *338*, 241–249. https://doi.org/10.1016/j.jhazmat.2017.05.022

Singha, N. R., Mahapatra, M., Karmakar, M., Dutta, A., Mondal, H., & Chattopadhyay, P. K. (2017). Synthesis of guar gum- *g* -(acrylic acid- *co* -acrylamide- *co* -3-acrylamido propanoic acid) IPN *via in situ* attachment of acrylamido propanoic acid for analyzing superadsorption mechanism of Pb(II)/Cd(II)/Cu(II)/MB/MV. *Polymer Chemistry*, *8*(44), 6750–6777. https://doi.org/10.1039/C7PY01564J

Şolpan, D., & Torun, M. (2005). Investigation of complex formation between (sodium alginate/acrylamide) semi-interpenetrating polymer networks and lead, cadmium, nickel ions. *Colloids and Surfaces A: Physicochemical and Engineering Aspects*, *268*(1–3), 12–18. https://doi.org/10.1016/j.colsurfa.2005.04.035

Şolpan, D., Torun, M., & Güven, O. (2008). The usability of (sodium alginate/acrylamide) semi-interpenetrating polymer networks on removal of some textile dyes. *Journal of Applied Polymer Science*, *108*(6), 3787–3795. https://doi.org/10.1002/app.27945

Sukriti, Sharma, J., Chadha, A. S., Pruthi, V., Anand, P., Bhatia, J., & Kaith, B. S. (2017). Sequestration of dyes from artificially prepared textile effluent using RSM-CCD optimized hybrid backbone based adsorbent-kinetic and equilibrium studies. *Journal of Environmental Management*, *190*, 176–187. https://doi.org/10.1016/j.jenvman.2016.12.065

Sun, D., Zheng, Z.-S., Liu, M.-Q., Li, B.-B., Huang, F., & Li, D.-Y. (2017). Structuring and characterization of a novel microporous PVDF membrane with semi-interpenetrating polymer

networks for vacuum membrane distillation. *Polymer Engineering & Science*, *57*(12), 1311–1321. https://doi.org/10.1002/pen.24514

Taktak, F. F., & Özyaranlar, E. (2022). Semi-interpenetrating network based on xanthan gum-cl-2-(N-morpholinoethyl methacrylate)/titanium oxide for the single and binary removal of cationic dyes from water. *International Journal of Biological Macromolecules*, *221*, 238–255. https://doi.org/10.1016/j.ijbiomac.2022.08.139

Tanan, W., Panpinit, S., & Saengsuwan, S. (2021). Comparison of microwave-assisted and thermal-heated synthesis of P(HEMA-co-AM)/PVA interpenetrating polymer network (IPN) hydrogels for Pb(II) removal from aqueous solution: Characterization, adsorption and kinetic study. *European Polymer Journal*, *143*, 110193. https://doi.org/10.1016/j.eurpolymj.2020.110193

Tian, E. L., Zhou, Huan., Ren, Y. W., mirza, Zakaria. a., Wang, X. Z., & Xiong, S. W. (2014). Novel design of hydrophobic/hydrophilic interpenetrating network composite nanofibers for the support layer of forward osmosis membrane. *Desalination*, *347*, 207–214. https://doi.org/10.1016/j.desal.2014.05.043

Toledo, P. V. O., Limeira, D. P. C., Siqueira, N. C., & Petri, D. F. S. (2019). Carboxymethyl cellulose/poly(acrylic acid) interpenetrating polymer network hydrogels as multifunctional adsorbents. *Cellulose*, *26*(1), 597–615. https://doi.org/10.1007/s10570-018-02232-9

Üzüm, Ö. B., & Karadağ, E. (2012). Behavior of semi IPN hydrogels composed of PEG and AAm/SMA copolymers in swelling and uptake of Janus Green B from aqueous solutions. *Journal of Applied Polymer Science*, *125*(5), 3318–3328. https://doi.org/10.1002/app.36586

Waheed, A., Baig, U., & Ansari, M. A. (2022). Fabrication of CuO nanoparticles immobilized nanofiltration composite membrane for dye/salt fractionation: Performance and antibiofouling. *Journal of Environmental Chemical Engineering*, *10*(1), 106960. https://doi.org/10.1016/j.jece.2021.106960

Wang, C., Wang, Z., Yang, F., & Wang, J. (2021). Improving the permselectivity and antifouling performance of reverse osmosis membrane based on a semi-interpenetrating polymer network. *Desalination*, *502*, 114910. https://doi.org/10.1016/j.desal.2020.114910

Wang, J., Han, Y., Zhao, G., & Chen, X. (2016). Ion-imprinted interpenetrating network gels for solid-phase extraction of heavy metal ions. *International Journal of Environmental Analytical Chemistry*, *96*(14), 1341–1355. https://doi.org/10.1080/03067319.2016.1251913

Wang, J., & Li, J. (2015). One-pot Synthesis of IPN Hydrogels with Enhanced Mechanical Strength for Synergistic Adsorption of Basic Dyes. *Soft Materials*, *13*(3), 160–166. https://doi.org/10.1080/1539445X.2015.1047957

Wang, J., Liu, F., & Wei, J. (2011). Enhanced adsorption properties of interpenetrating polymer network hydrogels for heavy metal ion removal. *Polymer Bulletin*, *67*(8), 1709–1720. https://doi.org/10.1007/s00289-011-0579-8

Wang, J., Zhang, Y., Wang, L., Feng, R., & Zhang, F. (2018). Competitive Adsorption removal of Congo red and Rhodamine B over alkaline membrane from in situ polymerization of Gemini cationic molecule. *Journal of the Iranian Chemical Society*, *15*(1), 141–152. https://doi.org/10.1007/s13738-017-1217-7

Wang, R., Jiang, X., He, A., Xiang, T., & Zhao, C. (2015). An in situ crosslinking approach towards chitosan-based Semi IPN hybrid particles for versatile adsorptions of toxins. *RSC Advances*, *5*(64), 51631–51641. https://doi.org/10.1039/C5RA04638F

Wang, S., Chen, X., Li, B., Shi, X., Shi, Y., Wang, J., Pan, J., & Wan, D. (2022). One-step selective separation and catalytic transformation of an organic pollutant from pollutant mixture via a thermo-responsive Semi IPN/PVDF@Pd bilayer composite membrane. *Separation and Purification Technology*, *286*, 120493. https://doi.org/10.1016/j.seppur.2022.120493

Wang, X., Hou, H., Li, Y., Wang, Y., Hao, C., & Ge, C. (2016). A novel Semi IPN hydrogel: Preparation, swelling properties and adsorption studies of Co (II). *Journal of Industrial and Engineering Chemistry*, *41*, 82–90. https://doi.org/10.1016/j.jiec.2016.07.012

Wang, X., Li, Z., Wu, Y., Guo, H., Zhang, X., Yang, Y., Mu, H., & Duan, J. (2021). Construction of a Three-Dimensional Interpenetrating Network Sponge for High-Efficiency and Cavity-Enhanced Solar-Driven Wastewater Treatment. *ACS Applied Materials & Interfaces*, *13*(9), 10902–10915. https://doi.org/10.1021/acsami.0c21690

Wu, L., Shi, M., Guo, R., & Dong, W. (2022). Development of a novel pullulan/polydopamine composite hydrogel adsorbent for dye removal. *Colloids and Surfaces A: Physicochemical and Engineering Aspects*, *652*, 129632. https://doi.org/10.1016/j.colsurfa.2022.129632

Xu, Z., Wu, K., Luo, H., Wang, Q., Zhang, T. C., Chen, X., Rong, H., & Fang, Q. (2022). Electro-responsive SEMI-IPN hydrogel with enhanced responsive property for forward osmosis desalination. *Journal of Applied Polymer Science*, *139*(7), 51650. https://doi.org/10.1002/app.51650

Yilmaz, E., Guzel Kaya, G., & Deveci, H. (2019). Removal of methylene blue dye from aqueous solution by semi-interpenetrating polymer network hybrid hydrogel: Optimization through Taguchi method. *Journal of Polymer Science Part A: Polymer Chemistry*, *57*(10), 1070–1078. https://doi.org/10.1002/pola.29361

Zendehdel, M., Barati, A., Alikhani, H., & Hekmat, A. (2010). *Removal of methylene blue dye from wastewater by adsorption onto semi-inpenetrating polymer network hydrogels composed of acrylamide and acrylic acid copolymer and polyvinyl alcohol*.

Zhai, X., Chen, X., Shi, X., Wang, S., Wang, S., Wu, Q., Ma, Y., Wang, J., Wan, D., & Pan, J. (2022). Simultaneously enhancing purification, catalysis and in situ separation in a continuous cross-flow catalytic degradation process of multi-component organic pollutants by a double-layer PVDF composite membrane. *Journal of Environmental Chemical Engineering*, *10*(2), 107160. https://doi.org/10.1016/j.jece.2022.107160

Zhang, M., Li, Y., Yang, Q., Huang, L., Chen, L., & Xiao, H. (2018). Adsorption of methyl violet using pH- and temperature-sensitive cellulose filament/poly(NIPAM-co-AAc) hybrid hydrogels. *Journal of Materials Science*, *53*(16), 11837–11854. https://doi.org/10.1007/s10853-018-2342-0

Zhang, P., Yin, J., & Jiang, X. (2014). Hyperbranched Poly(ether amine) (hPEA)/Poly(vinyl alcohol) (PVA) Interpenetrating Network (IPN) for Selective Adsorption and Separation of Guest Homologues. *Langmuir*, *30*(48), 14597–14605. https://doi.org/10.1021/la502869n

Zhao, S., Zhou, F., Li, L., Cao, M., Zuo, D., & Liu, H. (2012). Removal of anionic dyes from aqueous solutions by adsorption of chitosan-based Semi IPN hydrogel composites. *Composites Part B: Engineering*, *43*(3), 1570–1578. https://doi.org/10.1016/j.compositesb.2012.01.015

Zhao, Z., Huang, Y., Wu, Y., Li, S., Yin, H., & Wang, J. (2021). α-ketoglutaric acid modified chitosan/polyacrylamide semi-interpenetrating polymer network hydrogel for removal of heavy metal ions. *Colloids and Surfaces A: Physicochemical and Engineering Aspects*, *628*, 127262. https://doi.org/10.1016/j.colsurfa.2021.127262
